# Changes in Systemic Regulatory T Cells, Effector T Cells, and Monocyte Populations Associated With Early-Life Stunting

**DOI:** 10.3389/fimmu.2022.864084

**Published:** 2022-06-02

**Authors:** Zo Andriamanantena, Fanirisoa Randrianarisaona, Maheninasy Rakotondrainipiana, Prisca Andriantsalama, Ravaka Randriamparany, Rindra Randremanana, Frédérique Randrianirina, Sophie Novault, Darragh Duffy, François Huetz, Milena Hasan, Matthieu Schoenhals, Philippe J. Sansonetti, Pascale Vonaesch, Inès Vigan-Womas, Afribiota Investigators, Laurence Barbot-Trystram

**Affiliations:** ^1^ Immunology of Infectious Diseases Unit, Institut Pasteur de Madagascar, Antananarivo, Madagascar; ^2^ Epidemiology and Clinical Research Unit, Institut Pasteur de Madagascar, Antananarivo, Madagascar; ^3^ Clinical Biology Center, Institut Pasteur de Madagascar, Antananarivo, Madagascar; ^4^ Cytometry and Biomarkers Unit of Technology and Service, Université de Paris, Institut Pasteur, Paris, France; ^5^ Translational Immunology Lab, Department of Immunology, Université de Paris, Institut Pasteur, Paris, France; ^6^ Antibodies in Therapy and Pathology Unit, Institut Pasteur, Paris, France; ^7^ Institut Pasteur, Molecular Microbial Pathogenesis Unit, Paris, France

**Keywords:** stunting, environmental enteric dysfunction, flow cytometry, monocytes, regulatory T cells, Madagascar, systemic immune cells

## Abstract

Stunting and environmental enteric dysfunction (EED) may be responsible for altered gut and systemic immune responses. However, their impact on circulating immune cell populations remains poorly characterized during early life. A detailed flow cytometry analysis of major systemic immune cell populations in 53 stunted and 52 non-stunted (2 to 5 years old) children living in Antananarivo (Madagascar) was performed. Compared to age-matched non-stunted controls, stunted children aged 2-3 years old had a significantly lower relative proportion of classical monocytes. No significant associations were found between stunting and the percentages of effector T helper cell populations (Th1, Th2, Th17, Th1Th17, and cTfh). However, we found that HLA-DR expression (MFI) on all memory CD4^+^ or CD8^+^ T cell subsets was significantly lower in stunted children compared to non-stunted controls. Interestingly, in stunted children compared to the same age-matched non-stunted controls, we observed statistically significant age-specific differences in regulatory T cells (Treg) subsets. Indeed, in 2- to 3-year-old stunted children, a significantly higher percentage of memory Treg, whilst a significantly lower percentage of naive Treg, was found. Our results revealed that both innate and adaptive systemic cell percentages, as well as activation status, were impacted in an age-related manner during stunting. Our study provides valuable insights into the understanding of systemic immune system changes in stunted children.

## Introduction

Stunted child growth is a consequence of chronic undernutrition and remains one of the most important global health problems worldwide (1). In 2019, an estimated 144 million children under 5 years of age are stunted, with the highest burden being observed in Sub-Saharan African and South-East Asian countries (1). Despite decades-long efforts to treat and reduce malnutrition through nutritional rehabilitation programs, complementary feeding interventions have been shown to reduce stunting only by one-third (2).

Several studies have shown that children living in low-to-middle income countries (LMICs) countries have an altered immune response to several live-attenuated vaccines, especially when orally administered (3–8). Various factors may explain this underperformance: i) higher titers of IgA antibodies in the breastmilk from mothers in LMICs compared to the mothers in high-income countries, which may inhibit the viral replication of lived-attenuated vaccines in the infant’s gut and would potentially impair their efficacy in eliciting an immune response (9–12); ii) pre-existing vitamin A deficiency which has been described as impairing gastrointestinal immunity (13); and iii) the presence of environmental enteric dysfunction (EED) (14–18).

EED is considered to be a subclinical disorder of the small intestine without overt diarrhea. Repeated exposure to a highly microbiologically contaminated environment and sustained infectious gastroenteritis are hypothesized to impair the gut structure and function, leading to a hyperimmune inflammatory state (17, 19, 20). Currently, existing studies to improve our understanding of the relationship between EED-related undernutrition and oral vaccine failure are sparse. However, there is growing evidence that undernutrition can persistently compromise children’s immune defenses against infection despite food rehabilitation (8, 21).

The identification of possible abnormalities in circulating immune cells in stunted children compared to non-stunted controls is critical for understanding aberrant immune responses, such as reduced vaccine responsiveness and/or increased susceptibility to infection in children living in LMICs. Moreover, specific phenotypes or proportions of blood immune cells, in combination with other biological biomarkers such as alpha1-antitrypsin (AAT), calprotectin, anti-flagellin, or anti-LPS (lipopolysaccharide) immunoglobulins could prove to be promising diagnostic biomarkers for EED (22, 23).

In this study, we hypothesized that stunted children may display circulating immune cells immunophenotypic abnormalities. To test this hypothesis, we investigated blood cell populations in children aged 2 to 5 years living in Antananarivo, the capital city of Madagascar. We studied innate and adaptive immune cells including monocytes, neutrophils, B cells, and T cells subsets. As non-genetic factors and the environment are responsible for 50% to 80% of the variability observed in circulating immune cells (24, 25), we also considered anemia, systemic inflammation (C-reactive protein (CRP) measurement), and asymptomatic pathogen carriage as covariables that may affect immune cell populations (26–28). Fecal markers of intestinal inflammation and barrier disruption (alpha1-antitrypsin (AAT) and calprotectin) have been reported as potential biomarkers for EED and are also associated with subsequent linear growth delay (29). Therefore, we also analyzed the potential association of these biomarkers with stunting and the immune blood cell populations.

## Materials and Methods

### Ethics Approval and Consent to Participate

The study protocol of AFRIBIOTA was approved by the Malagasy National Biomedical Research Ethics Committee of the Ministry of Public Health (55/MSANP/CE, May 19, 2015) and the Institutional Review Board of the Institut Pasteur, Paris (2016-06/IRB). All participants received oral and written information about the study, and the legal representatives of the children provided written consent to participate. A copy of the written consent is available for review by the Editor of this journal.

### Study Participants and Sample Collection

This study was nested within the AFRIBIOTA project (30) implemented in Madagascar and conducted in compliance with the principles of the Declaration of Helsinki. Community and hospital-based recruitment of children occurred between November 2016 and March 2018. Community recruitment was conducted in two of the poorest neighborhoods of Antananarivo (Andranomanalina Isotry and Ankasina), Madagascar. Eligible children aged 2 to 5 years old who were seeking care at the “Centre Hospitalier Universitaire Mère-Enfant de Tsaralalàna (CHUMET),” in the Pediatric Surgery Service of the “Hôpital Joseph Ravoahangy Andrianavalona (HJRA)” or in the “Centre de Santé Maternelle et Infantile de Tsaralalàna (CSMI)” and who met the inclusion and exclusion criteria were also invited to participate in the study as hospital-recruited children. Exclusion criteria were HIV-positive status, presence of acute malnutrition or any other severe disease. Included children were admitted to the hospital for sample collection and anthropometric measurement.

For this immunological study, we first selected children with a complete epidemiological data set (no missing data for age, sex, anthropometric measurements, and sampling date), sufficient blood volume (4 mL) at the collection time, and properly cryopreserved white blood cell samples. Stunted and control children were subsequently matched according to age in years, gender, neighborhood, and season at inclusion (dry or wet season). At the end of this selection process, the final cohort included 105 children (52 controls and 53 children with stunting) in this analysis. A flowchart of the children included in the analysis is shown in **Supplementary Figure S1**.

Four milliliters (mL) of whole blood were collected from each child, in a Lithium-heparin coated vacutainer tube by standard venipuncture. Blood samples were maintained at room temperature (RT, 18°C –25°C) until processing. Tracking procedures were established to ensure delivery to Institut Pasteur de Madagascar within 2 to 3 hours of blood draw. A complete blood count was performed (Sysmex^®^ XT-2000i Hematology Analyzer) immediately upon reception of samples.

Methods for stool sample collection and examination for the presence of helminth eggs, helminth larvae, and protozoan infestation using Kato–Katz smear, formol-ethyl acetate-concentration methods (MIF), and real-time PCR have been previously described (31). Real-time PCR was carried out on a CFX 96 Real Time system (BIO-RAD, France) for the following parasites: *A. lumbricoides, G. intestinalis, E. histolytica, Cryptosporidium parvum*, and *Isospora belli*, and the microsporidia species *Enterocytozoon bieneusi* and *Encephalitozoon* spp. *Schistosoma mansoni* eggs were not detected in stool samples of children included in this cohort.

### Isolation and Storage of White Blood Cells for Immunophenotyping

White blood cells (WBCs) were isolated using a red blood cell lysis solution (BD Biosciences, ref. 349202). Briefly, after plasma collection, blood cells (collected into Li-heparin) were washed by mixing fresh whole blood and Phosphate-Buffered Saline (PBS, GIBCO/Life Technologies, ref. 14200-067) at a 1:1 ratio, followed by centrifugation at 500g for 5 min at room temperature (RT). Red blood cells were lysed, and WBCs fixed in 1X FACS Lysis solution (BD Biosciences, ref. 349202) for 15 min at RT protected from light as recommended by the manufacturer. WBCs were thereafter spun down for 5 min at 500g and the supernatant was discarded. WBCs were then washed twice in 2 ml of PBS and aliquots were cryopreserved in PBS 1X supplemented with 50% FCS (Fetal Calf Serum, GIBCO/Life Technologies, ref. 10270) and 10% DMSO (dimethyl sulfoxide, GIBCO/Life Technologies, ref. D2650) until used.

### WBCs Thawing and Staining Protocol for Cytometric Analysis

WBCs were thawed rapidly in a 37°C water bath and resuspended in 300µl of PBS 1X. After centrifugation for 5 min at 500g, the supernatant was discarded, and cells were resuspended in 100 µl of PBS 1X. Cells were stained with four different 8-color flow cytometry panels for the characterization of immune blood cell populations. The antibody panels, based on the panels developed by the “Milieu Interieur Consortium” (32) are summarized in **Supplementary Table S1**. The “lineage panel” enabled the detection of major immune cell populations, including B cells (CD45^+^CD19^+^) and T cells (CD45^+^CD16^-^CD3^+^), NK cells, monocytes, and neutrophils (**Supplementary Figure S2**). Different subsets of T cells were classified in the “T cell” panel based on the relative levels of expression of CD27 and CD45RA: naïve T cells (TN, CD45RA^+^CD27^+^), central memory T cells (TCM, CD45RA^-^ CD27^+^), effector memory T cells (TEM, CD45RA^-^CD27^-^), and effector memory T cells CD45RA^+^ (TEMRA, CD45RA^+^CD27^-^). We also assessed the expression of CCR7 (MFI) as this chemokine receptor has been used to define TN and TCM. Activation status was determined by the Major histocompatibility complex (MHC) II cell surface receptor (HLA-DR) expression (MFI) (**Supplementary Figure S3**). CCR7 or CD27 are two markers used for naïve and memory T cells characterization. In our analyses, we found only weak signals for CCR7 antibody, which did not allow us to distinguish the positive and negative cell populations more accurately. Therefore, we chose CD27 surface staining to characterize TN/TCM/TEM/TEMRA subpopulations as this marker is a good substitute for CCR7 (33). The “T helper cells” (Th) panel enabled the detection of different Th cell subsets: classical T-helper 1, (Th1, CD183^+^CCR6^-^), Th17 (CD183^-^CCR6^+^), Th1Th17 subsets (CD183^+^CCR6^+^CD183^+^CD194^+^), Th2 (CD183^-^CCR6^-^CD294^+^CXCR5^-^), and circulating follicular helper T cells (cTfh, CD294^-^CXCR5^+^) (**Supplementary Figure S4**). The proportions of regulatory T cells (Tregs) were defined by CD3^+^CD4^+^CD25^+^CD127^-^ in the “Treg panel”. Based on CD45RA and HLA-DR expression levels, Treg cells subsets were characterized as naïve (CD45RA^+^HLA-DR^-^), memory (CD45RA^-^HLA-DR^-^), and activated Treg (CD45RA^-^HLA-DR^+^). Relative mean fluorescence intensity (MFI) of the inducible T cell co-stimulator (ICOS, CD278) was also calculated in these Treg subsets (**Supplementary Figure S5**). After the addition of antibody cocktails to the WBC solution, samples were briefly vortexed and incubated for 20 min at RT protected from light. Cells were centrifugated for 5 min at 500g and resuspended in 240 μl of PBS 1X. The entire tube (all events) was immediately acquired using acoustic focusing Attune™ NxT Flow Cytometer (Thermo Fisher Scientific, Waltham, MA, USA) and data were analyzed by FlowJo software (version 10.3., Treestar). Samples with less than 5000 intact singlet cells were discarded from the analysis. Thus, among the 105 samples initially selected, we were able to analyze 98 samples with the “lineage panel,” 102 samples with the “T cells panel,” 102 samples with the “Th cells panel,” and 103 samples with the “Treg panel” (**Supplementary Figure S1**).

Optimum concentrations for each antibody have been established by titration assay. Manual gating was done using Flowjo (TreeStar) software. Gates were individualized to participants based on events and to minimize bias introduced by manual repositioning of gates; magnetic gates were created for the brightest and most clearly defined antigens (e.g., CD4).

Instrument calibration was checked daily using the Attune Performance Tracking Beads (Thermo Fisher Scientific, ref. 4449754). Voltages were set such that the center of the histograms for the unstained control was around 10^2^ MFI units and the positive peaks for the single stain controls were around 10^4^ MFI units. The compensation matrix was calculated using unstained and single-stained cells. Cells stained as fluorescence-minus-one (FMO) controls were applied to antibodies that showed weak signals (e.g., CD294) and those for which it was difficult to define positive from negative cell populations.

To validate T cell data across the four panels, we calculated the coefficient of variation (CV) between T cell (CD3^+^) percentages for the “T cell” panel and “lineage” panel, and the CV between Th cell (CD4^+^) and Tc cells (CD8^+^) percentages between the four panels in each sample. Minimal inter-panel variabilities with intradonor CV below 15% between all the T cells subsets were observed (i.e., 2·5%, 3·9%, and 8·7% for CD3^+^, CD4^+,^ and CD8^+^ respectively (**Supplementary Figure S6A**). We also compared the CD4^+^ to CD8^+^ ratio between the four panels and found a strong correlation between the Th/Tc ratio as measured using a Spearman correlation test (rho > 0.90 for all) (**Supplementary Figure S6B**). Thus, cell percentage measurements were generally constant across all donors and panels, validating the robustness of the chosen approach.

### Assessment of Inflammatory Markers

Fecal calprotectin and alpha-1 antitrypsin (AAT) measurements were performed at “Hôpital de la Pitié-Salpêtrière,” Paris, France, and previously published (34). Briefly, stool samples were diluted 1:5 in 0.15M NaCl and vortexed vigorously until complete homogenization; the homogenate was then centrifuged, and the supernatant was collected for analysis. Calprotectin concentrations were assayed in duplicate by sandwich ELISA using a polyclonal antibody system (Calprest; Eurospital, Italy) according to the manufacturer’s instructions. Fecal AAT was measured using an immuno- nephelometric method adapted to the BN ProSpec system (Siemens, Germany) (35).

To assess serum C-reactive protein (CRP) and ferritin levels, venous blood was collected using EDTA vacutainer tubes and tests were performed within 4h after blood collection at the Clinical Biology Center of the Institut Pasteur de Madagascar. Ferritin was measured using Chemiluminescent Microparticle Immunoassay and corrected for systemic inflammation (36). CRP was assessed by Chemiluminescent Microparticle Immunoassay implemented on automated analyzers (Abbot Ilinity). Procedures were detailed in a previous study (37).

### Statistical Analysis

All analyses were conducted using R version 4.1.0 using vegan (38), adonis (39), ggplot2 (40), and mice (41) packages. We defined two outcomes: (1) relative proportions (%) and (2) mean fluorescence intensity (MFI). The primary objective of this study was to determine if the immune cell populations vary by stunting status in children aged 2 to 5 years. Our first parameter was the height-for-age (HAZ) z-score, using the WHO Global Database on Child Growth and Malnutrition z-score cut-off point of < −2 standard deviations (SDs) (42). Mann-Whitney and Spearman’s tests were conducted to assess the variation between relative proportions (%) and fluorescence (MFI) of cells related to children’s stunting status (stunted vs. non-stunted), age, sex, systemic inflammation (CRP), EED inflammation biomarkers (calprotectin and alpha-antitrypsin), anemia, and pathogen carriage. P-values obtained by the Mann-Whitney and Spearman’s tests for each cell subset were corrected by recalculating them using the Benjamini-Hochberg false discovery rate (FDR; p-value<0.05) correction method. The variables associated with variation of relative proportions or MFI (based on p < 0.20 in Mann-Whitney and Spearman’s test) were selected as the possible independent variables for multivariable analyses.

For multivariable analysis, we used the continuous version HAZ score, because it potentially contained more information than the dichotomized version (stunted/non-stunted). We ran multivariable linear regression analysis on completed data following multiple imputations of missing values. Missing values for outcomes were imputed with half the minimum of the values. Missing values for exposure were imputed by the mean for continuous variables and by logistic regression imputation for binary variables using mice package in R (Buuren, 2011). We have also checked the similarities in the distribution of original and imputed data using the same package (mice) in R. After controlling for age and sex, we further adjusted for the other potential confounders. Multicollinearity between covariates was examined by assessing the variance inflation factor (VIF), and only those with VIF < 10 were kept as variables in the final model. The R^2^ adjusted of the models, as well as the unstandardized regression coefficients (B), standard error (SE), and standardized regression coefficients (β), are reported in **Supplementary Tables S4-S7**.

Data structures of the cell population percentages in the four panels were explored with a non‐parametric method for multivariate analysis of variance (PERMANOVA test, adonis function in vegan) for each variable of interest (HAZ score, sex, age, parasites carriage, blood, and fecal inflammation biomarkers) to determine their contribution on the distribution (of percentages) of non-overlapped cell subpopulations in each panel (38, 39). The analysis was repeated and stratified into three age groups: children aged 2 to 3 years old (24-36 months), 3 to 4 years old (37-48 months), and 4 to 5 years old (49-60 months). All comparisons in the PERMANOVA tests were corrected for multiple testing using the Benjamini-Hochberg false discovery rate (FDR; p-value<0.05).

## Results

### Description of the Study Population

The 105 Malagasy children aged 2 to 5 years selected for this immunological study represent a subset of those enrolled in the AFRIBIOTA project (**Supplementary Figure S1**) (30). The main characteristics of the study participants of this analysis are summarized in **Table 1**. Based on their HAZ scores, individuals were grouped as stunted (n = 53) versus non-stunted (n = 52) and subsequently categorized according to their age (2-3, 3-4, and 4-5 years old). Male (51%) and female (49%) children were almost equally represented in stunted and non-stunted subgroups. In each age stratum, samples were relatively evenly split between male and female children (p-value = 0.22). All children included in this study had normal weight-for-height (WHZ) z-score, as children with acute malnutrition (WHZ Z-score < -2) at recruitment time or during the selection process were excluded from this study (**Table 1**).

**Table 1:** Study population.

Description	All	Non-stunted	Stunted	p-value
**Gender**	*(N= 105)*	*(N= 52)*	*(N=53)*	0.85
Male	54 (51%)	26 (50%)	28 (53%)	
Female	51 (49%)	26 (50%)	25 (47%)	
**Age**	*(N= 105)*	*(N= 52)*	*(N=53)*	0.91
Median (months, 1st -3d quantiles)	44.6 (36-52)	43.7 (35-51)	44.8 (36-52)	
2 years (n, %)	27 (25.7%)	14 (26.9%)	13 (24.5%)	
3 years (n, %)	37 (35.2%)	19 (36.5%)	18 (33.9%)	
4-5 years (n, %)	41 (39%)	19 (36.5%)	22 (41.5%)	
**Nutritional status**	*(N= 105)*	*(N= 52)*	*(N=53)*	
Median HAZ score (variance)	-2.02 (1.25)	-1.06 (0.32)	-3.05 (0.67)	**< 2.2e-16**
Median WHZ score (variance)	-0.26 (0.7)	-0.04 (0.73)	-0.31 (0.69)	0.61
**Anemia**	*(N= 105)*	*(N= 52)*	*(N=53)*	
Ferritin (µg/L)	25.8 (12-43)	27.1 (14-44)	20.9 (11-38)	0.34
Hemoglobin (g/100 mL serum)	11.5 (10.8-12.2)	11.6 (11-12)	11.4 (10-12)	0.17
Presence of anemia (hemoglobin level <11g/100 mL)	31 (29.3%)	13 (25%)	18 (33.3%)	0.4
**Inflammation biomarkers**	*(N=96)*	*(N= 47)*	*(N=49)*	
AAT-mg/g dry fecal weight	59.5 (34-150)	47 (30-72)	82 (45-114)	**0.006**
Calprotectin- μg/g dry fecal weight	445 (252-2679)	375 (191-534)	544 (158-1330)	**<0.001**
	*(N= 105)*	*(N= 52)*	*(N=53)*	
Elevated CRP (> 10 mg/L serum)	12 (11.4%)	4 (7.7%)	8 (15%)	0.36
**Complete Blood Count**	*(N= 105)*	*(N= 52)*	*(N=53)*	
Leucocytes number/mm3	9050 (7320-1140)	8830 (7320-11160)	9160 (7290-11020)	0.77
% Lymphocytes	43 (36-51)	42.2 (36-53)	44 (36-49)	0.83
% Monocytes	7.1 (6-8.5)	6.35 (5.6-8.4)	7.3 (6.1-8.5)	0.21
% Neutrophils	36 (28.8-45)	36.9 (29.8-45)	34.8 (28.5-44.7)	0.38
% Basophils	0.5 (0.3-0.7)	0.5 (0.4-0.7)	0.5 (0.3-0.7)	0.37
% Eosinophils	11 (5.6-15.3)	9.85 (5.43-12.9)	12.4 (7-17)	0.06
**Parasites carriage (PCR results), n (%)**	*(N= 86)*	*(N= 41)*	*(N=45)*	
*Giardia intestinalis*	68 (79%)	35 (85%)	33 (73%)	0.19
*Ascaris lumbricoides*	60 (70%)	25 (61%)	36 (78%)	0.1
*Trichuris trichiura* *	56 (65%)	24 (59%)	32 (71%)	0.26
*Enterocytozoon* spp.	26 (29%)	10 (24%)	15 (33%)	0.48
*Encephalocytozoon bieneusi*	13 (15%)	5 (12%)	7 (17%)	0.76
*Isospora belli*	19 (22%)	6 (15%)	13 (29%)	0.12
*Cryptosporidium parvum*	14 (16%)	7 (17%)	7 (16%)	1
*Entamoeba hystolytica*	9 (22%)	3 (7%)	6 (13%)	0.49

Values are expressed as median (1st and 3rd quantiles) for continuous variable, or as counts for categorical variables. Statistical analysis was performed using Mann Whitney test (continuous variable) or Fisher’s exact test (categorical variable). P-values indicate differences between non-stunted and stunted children. P-values <0.05 was considered statistically significant. Hemoglobin was adjusted by altitude (− 0.2 g/100 mL to account for the height above sea level). Parasite carriage was evaluated by qPCR and only presence of T. trichuria* was examined microscopically as the robustness of their eggs hampers optimal DNA isolation. AAT, alpha-1 antitrypsin; HAZ, height-for-age z-score; WHZ, weight-for-height z-score; CRP, C-reactive protein. Significant differences (p<0.05) are indicated in bold.

Two fecal inflammatory biomarkers (calprotectin and alpha-1 antitrypsin (AAT)) were analyzed to assess EED associated with stunting status. C-reactive protein (CRP) level was assessed as a systemic inflammation marker. We found that stunted children had higher levels of the mucosal inflammatory EED biomarkers, fecal AAT, and fecal calprotectin compared to non-stunted children (**Table 1**, Mann-Whitney test, p = 0.006 and p < 0.001 for AAT and calprotectin respectively). However, no significant association was found between the level of serum CRP, ferritin, or hemoglobin and stunting status (**Table 1**, Fisher’s exact test, p > 0.1). Protozoan and helminthic infections were common among all children with a predominance of *Giardia intestinalis* (79.3%) and *Ascaris lumbricoides* (70.1%). However, no significant association was found between stunted and not stunted children in terms of parasite and helminth carriage (**Table 1**).

### Monocyte Subsets Percentages Are Affected by Stunting Status and Age

Within CD45^+^ cells (lineage panel), we identified major immune cell populations, including B cells (CD19^+^CD16-CD3^-^), T cells (T helper/Th CD3^+^CD4^+^ and T cytotoxic/Tc CD3^+^CD8^+^), NK cells (CD3^-^CD56^+^), monocytes (CD16^+^SSC^low^), and neutrophils (CD16^hi^SSC^hi^). Classical, non-classical, and intermediate monocyte subsets were furthermore defined as CD14^+^CD16^low/int^, CD16^hi^CD14^low^, and CD16^hi^CD14^+^, respectively.

**Figure 1:**
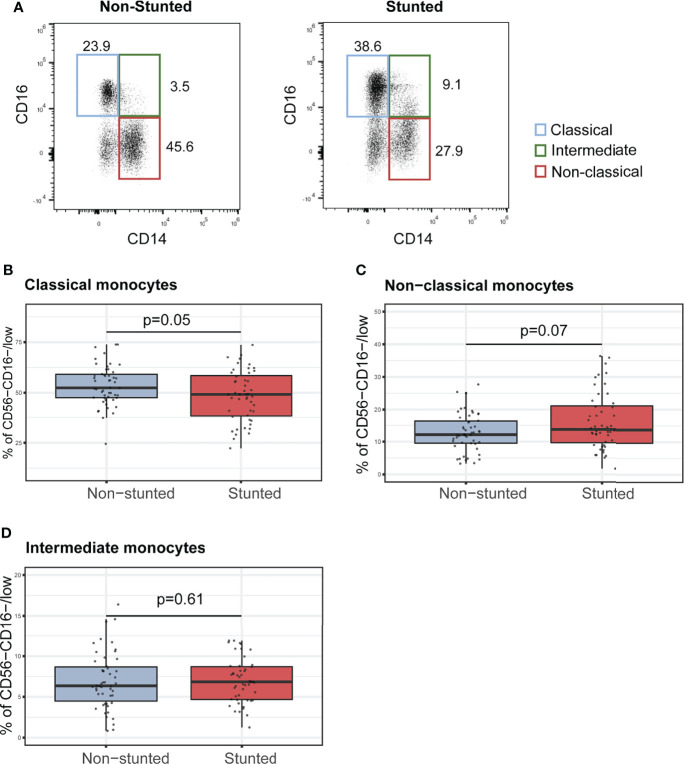
Characterization of monocyte subsets in non-stunted vs. stunted children. **(A)** Representative flow cytometry dot plots of CD14 and CD16 expression on monocyte subsets from non-stunted (left) and stunted (right) children. Classical, non-classical and intermediate monocyte subsets were defined as CD14^+^CD16^low/int^, CD16^hi^CD14^low,^ and CD16^hi^CD14^+,^ respectively. **(B–D)** Relationships between stunting status and the percentages of classical, non-classical, and intermediate monocyte populations. NS, non-stunted; S, stunted. Significance (p<0.05) was determined by Benjamini-Hochberg correction after Mann-Whitney test. *N* total = 98, N non-stunted = 50, *N* stunted=48.

For NK cells subsets, neutrophils, Th cells, Tc cells, and CD4^+^ to CD8^+^ cell ratio, no significant associations with stunting status were found (**Supplementary Tables S2-S3**). However, we observed that monocyte subclass percentages were associated with stunting status (**Figures 1A–C**). Indeed, children with stunting demonstrated a lower percentage of classical monocytes compared to non-stunted controls (**Figure 1B**, FDR-corrected, p = 0.05). In contrast, the relative percentages of non-classical monocytes tended to be higher in stunted children compared to controls (**Figure 1C**, FDR-corrected, p = 0.07). The relative percentage of intermediate monocytes differs between stunted and non-stunted children (**Figure 1D**, FDR-corrected, p=0.61). Multiple regression analysis adjusted for age, sex, *Encephalitozoon* carriage, serum CRP, and anemia was used to test if HAZ score predicted relative percentages of monocyte subtypes. The results indicated that the predictors explained 14% of the variance of the relative proportions of classical monocytes (adjusted R^2 =^ 0.14, p<0.002). HAZ score was found to be positively associated with classical monocytes (**Supplementary Table S4**, β = 0.21, p = 0.03). In the model predicting the non-classical monocytes relative proportions, the selected variables explained only 9% of the variance (adjusted R^2 =^ 0.09, p<0.02), And HAZ score tends to be negatively associated with non-classical monocytes (**Supplementary Table S4**, β = - 0.18, p = 0.07). In addition, the HAZ score was not found to be a significant predictor of the relative proportion of intermediate monocytes (**Supplementary Table S4**, β = 0.11, p = 0.26). Thus a 1-unit increase in HAZ score is associated with a lower proportion of classical monocytes by 0.21 SDs and a higher proportion of non-classical monocytes by 0.18 SDs.

### Stunting Is Related to Lower Surface Expressions of HLA-DR in Memory T Cell Subsets

**Figure 2:**
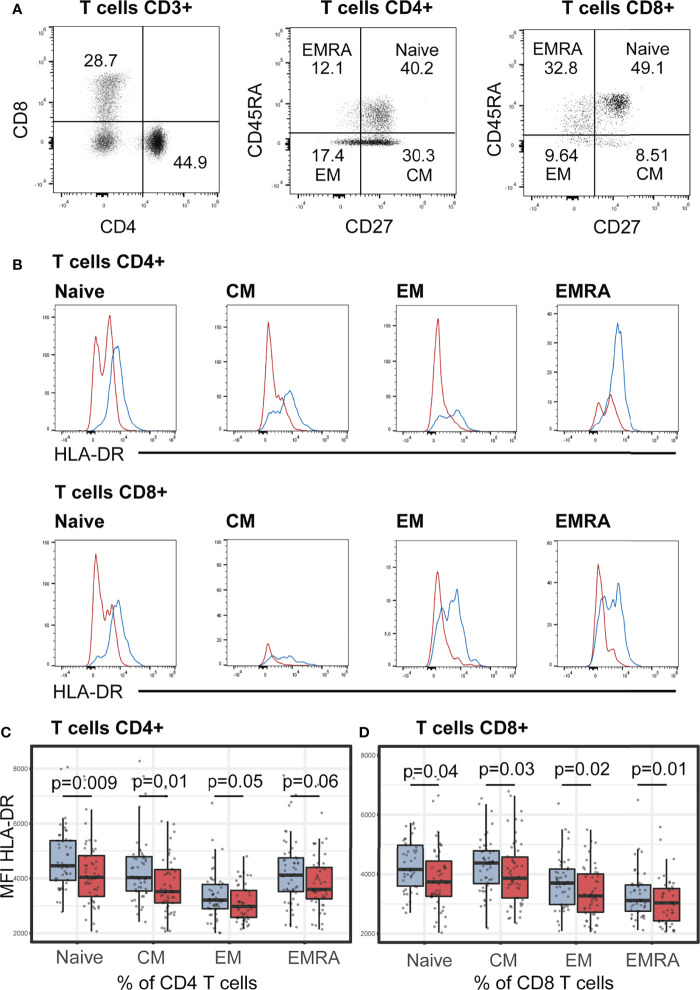
HLA-DR expression (MFI) on helper T (CD4+) and cytotoxic T cells (CD8+). **(A)** Representative dot plots of the gating strategy of T cells subsets based on their surface expression of CD27 and CD45RA markers. In each T cell subsets, naïve (CD27^+^CD45RA^+^), central memory (CM, CD27^+^CD45RA^-^), effector memory (EM, CD27^-^CD45RA^-^), and CD45RA^+^ effector memory (EMRA, CD27^-^CD45RA^+^) cells were defined. Representative dot plots from a non-stunted child are shown. **(B)** Representative histograms of HLA-DR expression on surface of naïve, CM, EM, EMRA T cells in stunted (red) versus non-stunted (blue) children. **(C, D)** Boxplot displaying the MFI values within all the T cell subsets in stunted (red) versus non-stunted (blue) children. Significance (p<0.05) was determined by Benjamini-Hochberg correction after Mann-Whitney test. *N* total= 101, N non-stunted = 50, *N* stunted=51.

We next investigated the memory phenotype of T helper (CD3^+^CD4^+^) and T cytotoxic (CD4^+^CD8^+^) cells classified based on their expressions of CD45RA and CD27 as naïve (TN, CD27^+^CD45RA^+^), central memory (TCM, CD27^+^CD45RA^-^), effector memory (TEM, CD27^-^CD45RA^-^), and CD45RA^+^ effector memory (TEMRA, CD27^-^CD45RA^+^) cells. No differences between the two groups of children (stunted vs. non-stunted) were observed for CD4^+^ and CD8^+^ memory T cells (**Supplementary Tables S2-S3**). Conversely, we found that HLA-DR expression (MFI) on all memory T cell subsets was significantly lower in stunted children compared to non-stunted controls: CD8^+^ naïve (FDR adjusted p = 0.01), CD8^+^CM (FDR adjusted p = 0.04), CD8^+^EM (FDR adjusted p = 0.03), CD8^+^EMRA (FDR adjusted p = 0.02), CD4^+^ naïve (FDR adjusted p = 0.009), CD4^+^CM (FDR adjusted p = 0.01), and CD4^+^EM (FDR adjusted p = 0.05) and CD4^+^EMRA (FDR adjusted p = 0.06, trend only) (**Figures 2A–D**).

We furthermore estimated the effect of stunting in the expression of HLA-DR (MFI) using multivariable linear regression analysis, adjusted for age and sex, and corrected with the two confounders selected in bivariate tests (Spearman correlation tests): *Encephalocytozoon* carriage and AAT. No associations between HAZ score and the variation of HLA-DR expression on CD4^+^ and CD8^+^ T cells were observed in any of the multivariable models (p > 0.05 for HAZ score).

### Th1/Th17 Cell Subsets Tend to be Lower in Stunted Children

T helper cell subsets were identified using different chemokine receptors: classical T-helper 1, Th1 (CD183^+^CCR6^-^), Th17 (CD183^-^CCR6^+^) and Th1Th17 subsets (CD183^+^CCR6^+^CD183^+^CD194^+^), Th2 (CD183^-^CCR6^-^CD294^+^CXCR5^-^) and circulating follicular helper T cells (cTfh, CD294^-^CXCR5^+^) (**Supplementary Figure S5**). In bivariate analyses (Mann-Whitney test), we found no significant associations between stunting status and the percentages of Th1, Th2, cTfh, nor Th17 cells (**Supplementary Table S2** and **Supplementary Figure S7**). Only the percentage of Th1Th17 was significantly lower in stunted children (**Supplementary Table S2** and **Supplementary Figure S7**, FDR adjusted p = 0.04). However, we did not observe a significant association between HAZ score and Th1Th17 percentage in the multiple linear regression analyses adjusted for age, sex, CRP, AAT, anemia, *Ascaris*, and *Encephalitozoon* carriage (β = 0.1, p = 0.35, adjusted R^2 =^ 0.13, p-value for model = 0.005 in linear regression, **Supplementary Table S6**). Thus, even though stunted children have a lower percentage of Th cell subsets compared to controls, the differences were not significant.

### Regulatory T Cell Subset Percentage Was Associated With Stunting, Age, and Asymptomatic *Isospora* Carriage

**Figure 3:**
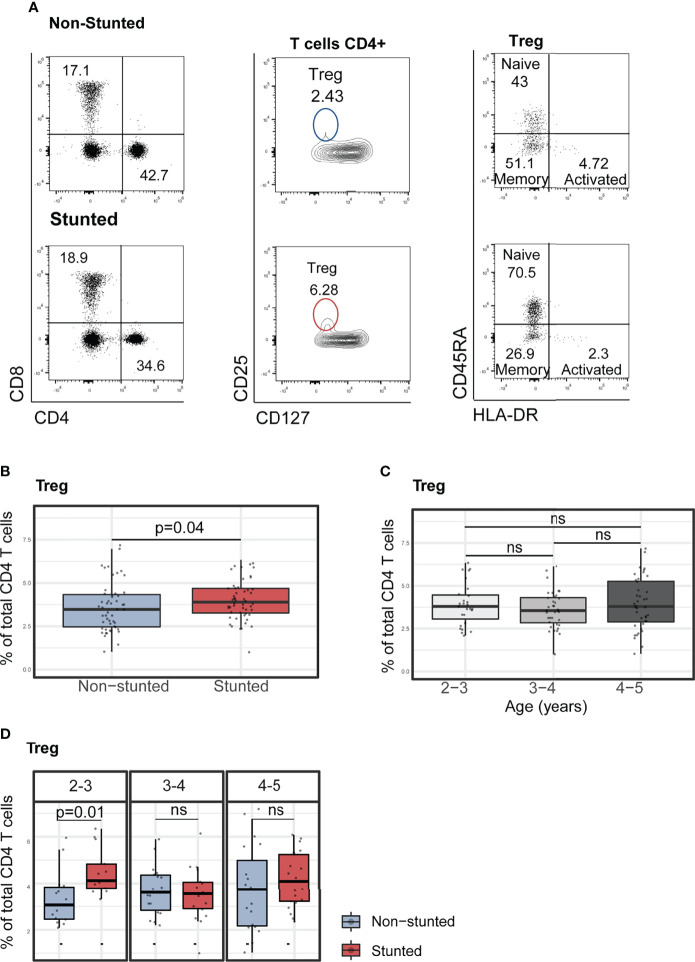
Characterization of peripheral blood regulatory T cells (Treg) and their subsets in non-stunted vs. stunted children. **(A)** Representative flow cytometry dot plots showing the differences of Treg cell percentages from one non-stunted vs. one stunted child. Regulatory T cells were gated in CD4+ cells based on their expression of CD25 and CD127 markers. Naïve, memory and activated Treg cell subsets were determined based on their expression of CD127 and HLA-DR. **(B)** Relationship between Treg cell percentages and stunting status and **(C)** child age in years. **(D)** Representative graphs of regulatory T cell percentages by age sub-groups (years) and stunting status. Significance (p<0.05) was determined by Benjamini-Hochberg correction after Mann-Whitney test. *N* total= 103, N non-stunted = 51, *N* stunted= 52. ns, not significant.

Finally, we determined the percentage of regulatory T cell (Treg, CD4^+^CD25^+^CD127^-^) populations in stunted and non-stunted children. Stunted children showed a higher percentage of Treg cells than non-stunted controls (**Figures 3A–B**, FDR adjusted p = 0.04). Relative proportions of Treg did not significantly differ between age subgroups (**Figure 3C**, Kruskal-Wallis test, p = 0.65). Interestingly, when we compared the percentages of Treg by age and stunting status, we observed that children aged 2 to 3 years old with stunting had a significantly higher relative percentage of Treg cells compared to age-matched controls (**Figure 3D**, FDR adjusted p = 0.01).

**Figure 4:**
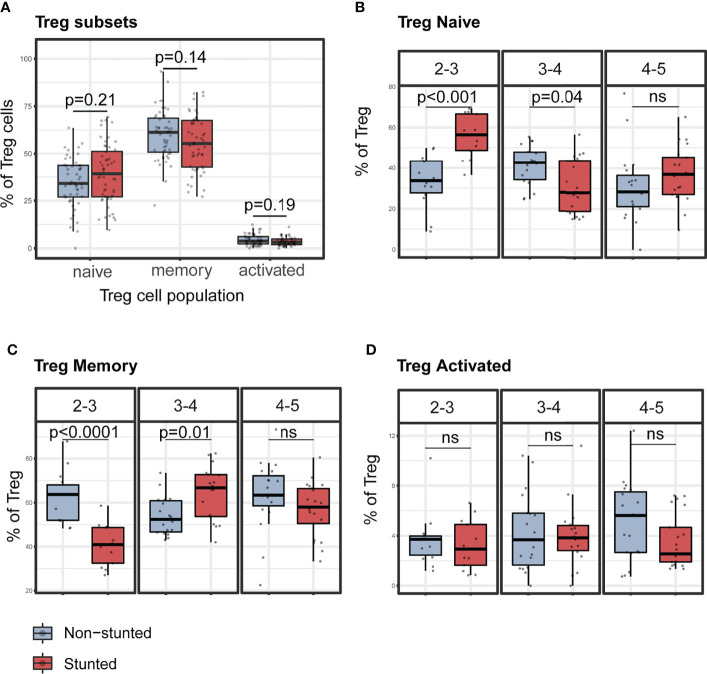
Representative graphs of the percentages of Treg cells subsets stratified by nutritional status and age sub-groups. **(A)** Percentages of regulatory T cell subsets children by stunting status. Percentages of naïve **(B)**, memory **(C)**, and activated **(D)** Treg cells by stunting status and age sub-groups (years). P-values were FDR corrected after Mann-Whitney test. *N* total= 103, N non-stunted = 51, *N* stunted= 52. ns, not significant.

Subsequently, we assessed the association between Treg percentages and HAZ score (adjusted for age and sex) by multivariable linear regression analysis. The covariates selected for the models included serum CRP, fecal AAT concentrations, *Ascaris*, and *Isospora*’s carriage (**Supplementary Table S7**). HAZ score was found to be negatively associated with lower relative percentages of Treg (β = -0.24, p = 0.02, adjusted R^2 =^ 0.17, p-value for model = 0.009). We further investigated the association between relative proportions of Treg cell subpopulations and stunting status. Treg cells were subdivided into naïve (CD4^+^CD25^+^CD127^-^CD45RA^+^HLA-DR^-^), memory (CD4^+^CD25^+^CD127^-^CD45RA^-^HLA-DR^-^), and activated Treg (CD4^+^CD25^+^CD127^-^CD45RA^-^HLA-DR^+^) subsets. In bivariate analyses with stunting status (Mann-Whitney test), no differences were observed within the naïve, memory, and activated Treg cell relative proportions (**Figure 4A**). However, the association with HAZ score was significant when investigated in a multivariable regression analysis of the naïve and memory Treg cells proportions (**Supplementary Table S7**). HAZ score was found to be negatively associated with relative percentage of naïve Treg (β = -0.23, p = 0.01, adjusted R^2 =^ 0.29, p for model <0.001) and positively associated with relative percentage of memory Treg (β = 0.21, p = 0.04, adjusted R^2 =^ 0.003, p for model =0.14). No significant associations between stunting status and relative percentage of activated Treg were identified in our model (**Supplementary Table S7**).

Percentages of naïve Treg were significantly higher in stunted children aged 2 to 3 years (FDR adjusted p < 0.001) and significantly lower in stunted children aged 3 to 4 years (FDR adjusted p = 0.04) compared to non-stunted, age-matched children (**Figure 4B**). Conversely, percentages of memory Treg were significantly lower in stunted children aged 2 to 3 years (FDR adjusted p < 0·001) and significantly higher in stunted children aged 3 to 4 years (FDR adjusted p = 0.02) compared to non-stunted, age-matched controls (**Figure 4C**). No significant differences were found in the activated Treg cell subset (**Figure 4D**).

### Association Between T Cell Populations, Environmental and Clinical Factors

**Figure 5:**
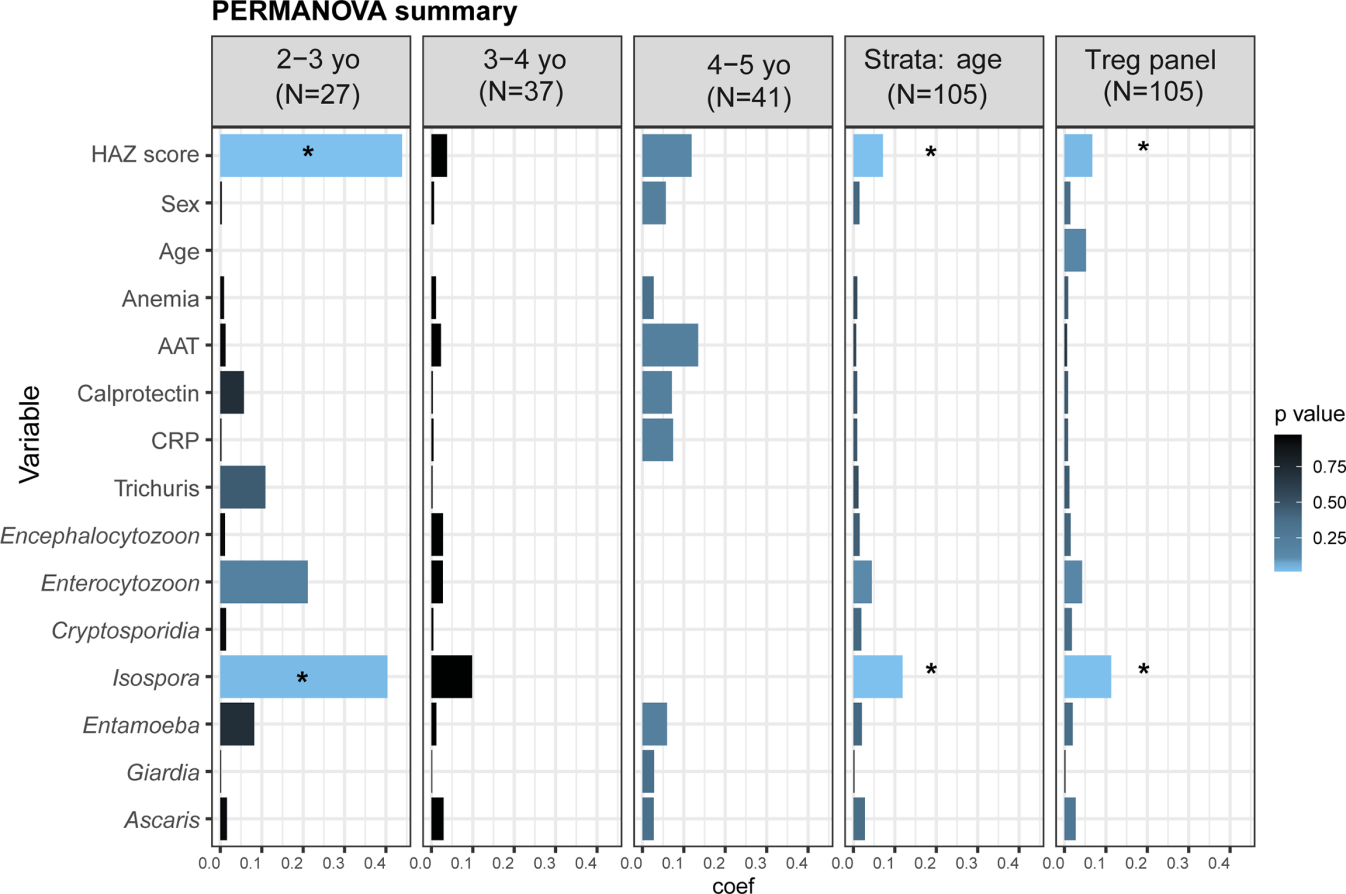
Distribution of cell percentages in the T reg panel. Summary of PERMANOVA analysis of the cell percentages in the full dataset, stratified by age and in each age category individually. The outcomes of the PERMANOVA analysis were the cells percentages in the “Treg panel”. As cell percentages are related to their “parents gate,” we analyzed the percentages of the five non-overlapped subpopulations: CD8^+^ Tc cells, CD4^+^CD25^-^CD127^+^ Th cells, Treg naïve (CD4^+^CD25^+^CD127^-^CD45RA^+^HLA-DR^-^), Treg memory (CD4^+^CD25^+^CD127^-^CD45RA^-^HLA-DR^-^), and Treg activated (CD4^+^CD25^+^CD127^-^CD45RA^-^HLA-DR^+^). The tested variables were as follows: HAZ score (height-for-age z-score); Age (child’s age in months); Anemia (presence or absence of anemia); AAT (fecal alpha-1 antitrypsin in mg/g dry weight); Calprotectin (fecal calprotectin in μg/g dry weight); CRP (serum C-reactive protein in mg/l); and parasite carriage. *Starred variables are significant with an FDR-corrected p<0.05. Each variable was tested individually in the PERMANOVA without other covariates. Coef: the coefficient of variance by PERMANOVA.

To identify environmental, clinical, or other factors that might influence the distribution of the measured cell phenotypes, we performed a PERMANOVA analysis of non-overlapping cell populations in each panel against selected variables putatively contributing to EED/and negatively affecting childhood nutritional status (**Supplementary Figure S8A–C** and **Figure 5**). Apart from HAZ score, age, sex, and anemia, we also assessed two putative EED inflammatory biomarkers (fecal AAT and calprotectin) and the systemic inflammatory marker (serum-CRP), along with asymptomatic parasites and protozoan carriage. For the “lineage,” “T cells,” and “T helper” panels, stunting status was not significantly associated with cell percentage distributions (**Supplementary Figure S8A–C**). Interestingly, stunting status and *Isospora* carriage were significantly associated with cell percentages in the “Treg” panel (**Figure 5**, partial variance explained 8%, FDR-corrected p = 0.02 for stunting and 15%, FDR-corrected p = 0.02 for *Isospora* carriage). To investigate the association with age in PERMANOVA, we repeated the analysis using age category stratification. Notably, impacts of HAZ score and *Isospora* carriage were significant in children aged 2 to 3 years. Stunting status explained 44% of the variance (FDR-corrected p = 0.01) while *Isospora* explained 40% of the variance (FDR-corrected p = 0.04) in the younger children. None of the inflammatory biomarkers nor the other parasites explained the variations in the differentiated cell percentages in older children (**Figure 5**).

## Discussion

Undernutrition, chronic exposure to pathogens, and immune response dysfunction are three components of a deleterious vicious cycle that greatly impact development during childhood. Indeed, it has been suggested that (i) undernutrition impairs innate and adaptive immune functions, increasing susceptibility to infectious diseases; (ii) in turn, energy and micronutrients are diverted to fight recurrent infections, resulting in impaired growth; and (iii) chronic exposure to various pathogens through unsanitary environments and/or leaky gut syndrome resulting from EED can lead to chronic systemic inflammation (8, 43). Yet, despite several studies (review in (8, 21, 44)), it is still not fully understood how systemic immune responses may be impacted during EED and stunting.

Monocytes are crucial in innate immunity for their high capacity to phagocyte, digest, process, and present antigens to lymphocytes. Phenotypically, monocytes can be distinguished by their expressions of CD14 and CD16. The classical monocytes (CD14^hi^CD16^-^) are known to be the main scavenger cells. Their major function is phagocytosis and the production of high levels of anti-inflammatory cytokines (IL-10) to counteract microbial infection (45). The intermediate monocytes (CD16^hi^CD14^+^) have the highest expression of genes associated with antigen presentation and seem to be the most efficient in presenting antigens to T cells (46, 47). The non-classical monocytes (CD16^hi^CD14^low^) are the effective producers of inflammatory cytokines (TNF-α and IL-1β) in response to microbial activation and are involved in FcR-mediated phagocytosis and adhesion (46). Our findings revealed that stunted children between 2 and 5 years of age tend to have a lower proportion of classical monocytes (CD14^hi^CD16^low/int^) and tend to have a higher percentage of non-classical monocytes (CD16^hi^CD14^low^). The impacts of undernutrition on monocyte evolution and their subpopulations have not been sufficiently investigated in humans (21). It has been shown that non-classical monocytes were increased in patients with inflammatory diseases such as rheumatoid arthritis (48). We suggest here that the higher percentage of the pro-inflammatory non-classical monocytes may regulate the immune response by enhancing cells proliferation, migration, and receptor expression in stunted children. Moreover, CD14^high^ monocytes constitute the main population from which intestinal macrophages are derived as they have a high ability for migration (49, 50). The lower proportion of systemic classical monocytes observed in stunting may be explained by the migration of these cells to the site of inflammation (for example, the intestinal mucosa in EED) (49, 51).

Helper CD4^+^ T cells (Th) and cytotoxic CD8^+^ T cells (Tc) are essential for adaptive immunity. Effector T cells play a significant role in the defense against pathogens, whereas regulatory T cells maintain homeostasis by limiting and/or suppressing effector T cells’ overactivity. No differences between the stunted vs. non-stunted children were found for CD4^+^ T cells and CD8^+^ T cells. We also observed that all effector helper T cell percentages (Th1, Th2, Th17, Th1Th17, and cTfh) tend to be lower in stunted children compared to non-stunted controls but these associations were not significant in our cohort of children. Several other studies in humans and mice have demonstrated a significant T-cell dysfunction in severe undernutrition or starvation (52, 53). In a recent study assessing the effects of undernutrition and *Mycobacterium tuberculosis* infection on baseline blood cell percentages, both percentages of CD4^+^ and CD8^+^ T cells were reported in children with low body mass index (54).

We also observed high circulating Treg cells in stunted compared to non-stunted children. The main role of Tregs is to maintain peripheral tolerance and suppress the function of effector T cells through suppressive cytokines (TGF-β and IL-10) (55). Increased percentages of circulating and intestinal regulatory T cells have also been reported in children with Crohn’s disease and ulcerative colitis, which share many biomarkers with EED (56–58).

The significantly higher proportion of systemic Treg cells in 2- to 3-year-old children could be associated with higher early exposure to microbes. Indeed, in Madagascar, this age period is generally associated with the beginning of weaning (37) and walking alone to explore their environment, which could be associated with increased exposure to an unsanitary environment. Extensive contact with this pathogen-contaminated environment in poor neighborhoods of Antananarivo was reflected by the high prevalence of parasitic infestation (more than 75% of the children were infected by at least one intestinal parasite) observed in all the children included in this study.

We also compared the proportion of Treg subpopulation subsets among all children, combining CD45RA and HLA-DR expression. Treg cells can be thymic-derived or differentiated from naïve CD4^+^ T cells exposed to non-self-antigens (59). Upon strong antigen stimulation, naïve Treg (CD45RA^+^HLA-DR^-^) are matured to highly suppressive activated Treg (CD45RA^-^HLA-DR^+^) and long-lasting memory Treg (CD45RA^-^HLA-DR^-^) (60). In our study, we have reported that the percentage of naïve Treg is significantly higher in young children aged 2 to 3 years, alongside a lower percentage of memory Treg. Interestingly, the relative proportions of these two Treg subpopulations are reversed among children aged 3 to 4 years. This may be explained by the fact that recurrent exposures to multiple antigens activate naïve Treg to effector memory Treg as children grow (61).

We also studied different factors that may modulate the immune system and have an impact on linear growth such as age and anemia (26), the known EED inflammatory biomarkers (fecal AAT and calprotectin, serum-CRP) (15, 29), and asymptomatic helminth and protozoan carriage (62–64). Interestingly, we found that the coccidian parasite *Isospora belli* appears to influence Treg subpopulations in some children (FDR-corrected p = 0.02 in PERMANOVA analysis). *Isospora belli* infection was shown to be associated with gastrointestinal disease and severe diarrhea in patients with immunodeficiency syndrome (AIDS) (65, 66). The number of children carrying the parasite was low (19/86, 22%). Thus, a study with a larger number of participants carrying the parasite would help to verify this finding. Altogether, these findings suggest that stunting may impact, in an age-specific manner, the proportion of naïve and memory regulatory T cell subsets in the peripheral blood of younger children.

Our results demonstrate that both innate and adaptive systemic cell proportions, as well as activation statuses, are impacted during chronic malnutrition in an age-related manner. However, our study has some limitations. As stunting status is a chronic disease, a longitudinal cohort study would be helpful to explore the temporal dynamics of peripheral immune cells. Larger sample size would also have been useful for a more powerful analysis of factors influencing the immune cell populations. Furthermore, the AFRIBIOTA study was performed both in Madagascar and in the Central African Republic. However, due to sample quality and availability, our study was restricted to the Malagasy children, preventing key comparisons with children living in another environmental context. We also selected the continuous version of the stunting status variable (i.e., HAZ score) in our multivariable analyses, as it gives more information than the dichotomized variable (stunted vs. non-stunted). This could introduce bias in the interpretation because, initially, we matched children according to the HAZ<-2 cutoff. Additionally, we recognize that circulating immune cells do not necessarily reflect the gut immune system. Simultaneous analysis of blood and mucosal immune cells in the same children would be very informative to better understand the relationship between mucosal and systemic immunity. It should be emphasized that mucosal biopsies were not possible in our study for practical and ethical reasons. However, to our knowledge, this is the first study that examines the phenotype of blood immune cells in stunted children, thus adding valuable data for a better understanding of immune changes in the context of chronic undernutrition.

In conclusion, our findings revealed that stunting may lead to a lower proportion of circulating classical monocytes, a lower HLA-DR expression (MFI) on all memory T cell subsets, and an age-specific significantly higher percentage of Tregs. Our data suggest that stunted children have a distinct circulating immune cell profile compared to non-stunted children, with both innate and adaptive blood cells being affected. For the first time, our results also suggest that the higher proportion of systemic Treg cells in 2- to 3-year-old children could be associated with early exploration of the highly pathogen-contaminated environments potentially requiring the immunosuppressive role of the Treg populations to counteract this stunting-related inflammatory process. We also illustrated the possible impact of *Isospora* carriage, a neglected enteric protozoan, in systemic Treg cell variations in a subgroup of children. Together, these findings bring new insights into the peripheral blood immune cell populations that could be associated with the susceptibility to infection in children with stunting.

## Data Availability Statement

The original contributions presented in the study are included in the article/**Supplementary Material**. Further inquiries can be directed to the corresponding author.

## Ethics Statement

The studies involving human participants were reviewed and approved by The Malagasy National Biomedical Research Ethics Committee of the Ministry of Public Health (55/MSANP/CE, May 19th, 2015) and the Institutional Review Board of the Institut Pasteur, Paris (2016-06/IRB). Written informed consent to participate in this study was provided by the participants’ legal guardian/next of kin.

## Afribiota Investigators (Group Authorship In Alphabetical Order)

**Laurence Barbot-Trystram**, Hôpital Pitié-Salpêtrière, Paris, France; **Robert Barouki**, Hôpital Necker- Enfants maladies, Paris, France; **Alexandra Bastaraud**, Institut Pasteur de Madagascar, Antananarivo, Madagascar; **Jean-Marc Collard**, Institut Pasteur de Madagascar, Antananarivo, Madagascar; **Maria Doria**, Institut Pasteur, Paris, France; **Darragh Duffy**, Institut Pasteur, Paris, France; **Serge Ghislain Djorie**, Institut Pasteur de Bangui, Bangui, Central African Republic; **Tamara Giles-Vernick**, Institut Pasteur, Paris, France; **Bolmbaye Privat Gondje**, Complexe Pédiatrique de Bangui, Bangui, Central African Republic; **Jean-Chrysostome Gody**, Complexe Pédiatrique de Bangui, Bangui, Central African Republic; **Milena Hasan**, Institut Pasteur, Paris, France; **Jean-Michel Héraud**, Institut Pasteur de Madagascar, Antananarivo, Madagascar; **Francis Allan Hunald**, Centre Hospitalier Universitaire Joseph Ravoahangy Andrianavalona (CHU-JRA), Antananarivo, Madagascar; **Nathalie Kapel**, Hôpital Pitié-Salpêtrière, Paris, France; **Jean-Pierre Lombart**, Institut Pasteur de Bangui, Bangui, Central African Republic; **Alexandre Manirakiza**, Institut Pasteur de Bangui, Bangui, Central African Republic; **Synthia Nazita Nigatoloum**, Complexe Pédiatrique de Bangui, Bangui, Central African Republic; **Laura Wegener Parfrey**, University of British Columbia, Vancouver, Canada; **Lisette Raharimalala**, Centre de Santé Materno-Infantile, Tsaralalana, Antananarivo, Madagascar; **Maheninasy Rakotondrainipiana**, Institut Pasteur de Madagascar, Antananarivo, Madagascar; **Rindra Vatosoa Randremanana**, Institut Pasteur de Madagascar, Antananarivo, Madagascar; **Harifetra Mamy Richard Randriamizao**, Centre Hospitalier Universitaire Joseph Ravoahangy Andrianavalona (CHU-JRA), Antananarivo, Madagascar; **Frédérique Randrianirina**, Institut Pasteur de Madagascar, Antananarivo, Madagascar; **Annick Lalaina Robinson**, Centre Hospitalier Universitaire Mère Enfant de Tsaralalana, Antananarivo, Madagascar; **Pierre-Alain Rubbo**, Institut Pasteur de Bangui, Bangui, République Centrafricaine; **Philippe Sansonetti**, Institut Pasteur, Paris, France; **Laura Schaeffer**, Institut Pasteur, Paris, France**; Ionela Gouandjika-Vassilache**, Instiut Pasteur de Bangui, Bangui, République Centrafricaine**; Pascale Vonaesch**, Institut Pasteur, Paris, France; **Sonia Sandrine Vondo**, Complexe Pédiatrique de Bangui, Bangui, Central African Republic; **Inès Vigan-Womas**, Institut Pasteur de Madagascar, Antananarivo, Madagascar.

## Author Contributions

PV, PS, and IV-W conceived and designed the study. PV, PS, IV-W, and ZA contributed to funding acquisition. RiR, PV, and IV-W were involved in project coordination and administration. MS, DD, FH, SN, MS, IV-W, and PV conceived the methodology. MR, PA, RaR, ZA, and RiR were involved in field work, recruitment of study participants, data, and sample collection. PV, MH, MS, and IV-W supervised the study. ZA, FrR, MR, PA, RaR, RiR, and PV performed data curation. ZA and FaR performed formal analysis. PV, MH, DD, MS, and IV-W validated and verified the underlying data. ZA, PV, and IV-W had access to data and contributed to data visualization. ZA, PV, and IV-W wrote the original draft. All authors reviewed, proofread, and edited revisions of the manuscript and had final responsibility for the decision to submit for publication.

## Funding

This project was funded by the Total Foundation, Institut Pasteur (PTR Immunohealth), Odyssey Re-Insurance company and the Petram Foundation. ZA was supported through a Calmette and Yersin traineeship grant from Institut Pasteur, and a Nutricia Research Foundation Fellowship Grant. PV was supported by an Early Postdoctoral Fellowship (Grant No. P2EZP3_152159), an Advanced Postdoctoral Fellowship (Grant No. P300PA_177876) as well as a Return Grant (Grant No. P3P3PA_17877) from the Swiss National Science Foundation, a Roux-Cantarini Fellowship (2016), a L’Oréal-UNESCO for Women in Science France Fellowship (2017) and a stipend through the Forschungsfonds of the University of Basel. The funders had no role in the study design or collection, analysis, and interpretation of data nor the writing of this manuscript.

## Conflict of Interest

The authors declare that the research was conducted in the absence of any commercial or financial relationships that could be construed as a potential conflict of interest.

## Publisher’s Note

All claims expressed in this article are solely those of the authors and do not necessarily represent those of their affiliated organizations, or those of the publisher, the editors and the reviewers. Any product that may be evaluated in this article, or claim that may be made by its manufacturer, is not guaranteed or endorsed by the publisher.
