# Supplementary material for: Changes in Systemic Regulatory T Cells, Effector T Cells, and Monocyte Populations Associated With Early-Life Stunting
Source: Front Immunol. 2022 Jun 2;13:864084. doi: 10.3389/fimmu.2022.864084 (PMC9202423; doi:10.3389/fimmu.2022.864084)
Supplement: Supplementary file 1 [file DataSheet_1.docx]

Supplementary Material

# 1 Supplementary Figures and Tables

## Supplementary Tables

| **Panel** | **Fluorochrome** | **Specificity** | **Clone (Supplier)** | **Reference** |
| --- | --- | --- | --- | --- |
| "Lineage" | eF450 | CD3 | SK7 (eBio) | 48-0036-42 |
|  | V500 | CD14 | M5E2 (BD) | 561391 |
|  | FITC | CD56 | NCAM16.2 (BD) | 345811 |
|  | PE | CD45 | HI30 (BD) | 555483 |
|  | PerCP-Cy5.5 | CD16 | 3G8 (BD) | 338440 |
|  | PE-Cy7 | CD8β | SIDI8BEE (eBio) | 25-5273-42 |
|  | APC | CD19 | SJ25C1 (BD) | 345791 |
|  | APC-H7 | CD4 | SK3 (BD) | 641398 |
| "T cells" | eF450 | CD3 | SK7 (eBio) | 48-0036-42 |
|  | V500 | HLA-DR | L243/G46-6 (BD) | 561224 |
|  | FITC | CD45RA | L48 (BD) | 335039 |
|  | PE | CD8α | BW135/80 (Miltenyi) | 130-104-130 |
|  | PerCP-eF710 | CD27 | O323 (eBio) | 46-0271-82 |
|  | PE-Cy7 | CD8β | SIDI8BEE (eBio) | 25-5273-42 |
|  | APC | CCR7 | FR11-11E8 (Miltenyi) | 130-093-624 |
|  | APC-H7 | CD4 | SK3 (BD) | 641398 |
| "T helper cells" | V450 | CCR4 (CD194) | 1G1 (BD) | 561123 |
|  | VioGreen | CRTH2 (CD294) | BM16 (Miltenyi) | 740189 |
|  | AF488 | CXCR5 (CD185) | RF8B2 (BD) | 558112 |
|  | PE | γδTCR | 11F2 (BD) | 333141 |
|  | PerCP-Cy5.5 | CCR6 (CD196) | 11A9 (BD) | 561752 |
|  | PE-Cy7 | CD8β | SIDI8BEE (eBio) | 25-5273-42 |
|  | APC | CXCR3 (CD183) | 1C6/CXCR3 (BD) | 560831 |
|  | APC-H7 | CD4 | SK3 (BD) | 641398 |
| "Regulatory T cells" | eF450 | ICOS | ISA-3 (eBio) | 48-9948-42 |
|  | FITC | CD45RA | L48 (BD) | 335039 |
|  | PE | CD25 | NA (Miltenyi) | 130-091-024 |
|  | PerCP-Cy5.5 | HLA-DR | L243/G46-6 (BD) | 339216 |
|  | PE-Cy7 | CD8β | SIDI8BEE (eBio) | 25-5273-42 |
|  | APC | CD127 | MB15-18C9 (Miltenyi) | 130-094-890 |
|  | APC-H7 | CD4 | SK3 (BD) | 641398 |

**Table S1:** List of Antibodies used for flow cytometry analysis.

**Table S2:** Percentages of circulating cells detected by flow cytometry. Results are expressed as median and interquartile range (IQR). Significant differences between stunted and non-stunted are depicted in bold letters (Mann-Whitney test).

| Variable name | Population name (Gating strategy) | Non-Stunted (%) | | Stunted (%) | | p-value |
| --- | --- | --- | --- | --- | --- | --- |
| "Lineage" panel | | | | | | |
| Leucocytes | CD45+ | 99 | (98-99) | 99 | (98-99) | 0.80 |
| B cells | CD45+ CD19+ | 7.30 | (5.9-8.9) | 8.35 | (5.6-12) | 0.10 |
| T cells | CD45+ CD19- CD3+ | 29.4 | (23-37) | 34.1 | (26-41) | 0.20 |
| Tc cells | CD45+ CD19- CD3+ CD8β+ | 34.8 | 31-40) | 35.5 | (31-39) | 0.71 |
| Th cells | CD45+ CD19- CD3+ CD4+ | 50 | (45-57) | 51.0 | (46-56) | 0.83 |
| NK cells | CD45+ CD19- CD3- | 6.23 | (4.8-8) | 7.45 | (4.6-9.7) | 0.45 |
| NK cells CD56high | CD45+ CD19- CD3- CD14- CD56+ | 1.86 | (1.2-3.4) | 1.78 | (1.3-2.7) | 0.95 |
| NK cells CD56low | CD45+ CD19- CD3- CD14- CD56high CD16low/- | 84.2 | (69-89) | 80.8 | (67-70) | 0.79 |
| Classical  monocytes | CD45+ CD19- CD3- CD56- CD14high CD16low/- | 52.4 | (48-59) | 49.2 | (39-58) | **0.05** |
| Intermediate monocytes | CD45+ CD19- CD3- CD56-CD14low CD16high | 6.36 | (4.5-8.7) | 6.84 | (4.6-8.7) | 0.61 |
| Non-classical monocytes | CD45+ CD19- CD3- CD56- CD14+ CD16high | 12.1 | (9.6-16.3) | 13.75 | (9.7-21.1) | 0.07 |
| Neutrophils | CD45+ CD19- CD3- CD56- CD16+ SSC+ | 99.1 | (98-100) | 98.9 | (97-100) | 0.82 |
| Ratio CD4:CD8β |  | 1.38 | (1.16-1.79) | 1.41 | (1.19-1.70) | 0.97 |
| "T cells" panel | | | | | | |
| T cells | CD3+ | 16.6 | (12-21) | 19.9 | (14-22) | 0.33 |
| Th cells | CD3+ CD4+ | 49.5 | (43-55) | 50.7 | (47-55) | 0.49 |
| T cells CD8α+ | CD3+ CD4+ CD8a+ | 1.95 | (1.5-2.4) | 2.04 | (1.6-3.2) | 0.23 |
| Th cells CM | CD3+ CD4+ CD27+ CD45RA- | 29.4 | (25-33) | 30.6 | (27-35) | 0.49 |
| Th cells EM | CD3+ CD4+ CD27- CD45RA- | 12.4 | (7.9-16) | 12.3 | (7.7-17) | 0.77 |
| Th cells EMRA | CD3+ CD4+ CD27- CD45RA+ | 2.45 | (1.1-3.9) | 1.86 | (0.9-3.3) | 0.22 |
| Th cells Naive | CD3+ CD4+ CD27+ CD45RA+ | 56.1 | (48-59) | 52.4 | (46-62) | 0.63 |
| Tc cells | CD3+ CD8β+ | 33.2 | (29-37) | 33.1 | (30-37) | 0.92 |
| Tc cells CM | CD3+ CD8β+ CD27+ CD45RA- | 14.8 | (9.8-19) | 13.5 | (11-19) | 0.79 |
| Tc cells EM | CD3+ CD8β+ CD27- CD45RA- | 9.97 | (6-16) | 9.76 | (6.9-18) | 0.41 |
| Tc cells EMRA | CD3+ CD8β+ CD27- CD45RA+ | 18.0 | (12-25) | 19.4 | (13-28) | 0.41 |
| Tc cells Naive | CD3+ CD8β+ CD27+ CD45RA+ | 53.2 | (44-62) | 53.1 | (39-64) | 0.59 |
| Ratio CD4:CD8β |  | 1.5 | (1.15-1.91) | 1.51 | (1.28-1.76) | 0.79 |
| "T helper cells" panel | | | | | | |
| TCR γδ+ | TCR γδ+ SSC low | 4.72 | (2.8-7.6) | 4.14 | (2.8-6.1) | 0.34 |
| TCR γδ- | TCR γδ- SSC low | 94.1 | (91-96) | 94.4 | (92-96) | 0.48 |
| Th cells | TCR γδ- SSC low CD4+ | 34.8 | (29-39) | 35.3 | (30-40) | 0.24 |
| Tc cells | TCR γδ- SSC low CD8β+ | 26 | (22-31) | 25.0 | (22-28) | 0.95 |
| Tc cells CCR6+ CD183- | TCR γδ- SSC low CD8β+ CCR6+ CD183- | 0.1 | (0.04-0.3) | 0.14 | (0.06-0.2) | 0.78 |
| Tc cells CCR6+ CD183+ | TCR γδ- SSC low CD8β+ CCR6+ CD183+ | 0.00 | (0-0.03) | 0.01 | (0-0.03) | 0.27 |
| Tc cells CCR6-CD183+ | TCR γδ- SSC low CD8β+ CCR6-CD183+ | 5.62 | (1.8-12) | 2.11 | (0.9-10) | 0.18 |
| Tc cells CCR6-CD183- | TCR γδ- SSC low CD8β+ CCR6-CD183- | 94.1 | (88-98) | 97.7 | (90-99) | 0.10 |
| Th1 cells | TCR γδ- SSC low CD4+ CCR6- CD183+ | 6.24 | (2.3-9.1) | 4.2 | (1.7-9.2) | 0.26 |
| Th2 cells | TCR γδ- SSC low CD4+ CCR6- CXCR5+ CD294- | 1.17 | (0.7-2) | 1.15 | (0.7-1.6) | 0.62 |
| Th17 cells | TCR γδ- SSC low CD4+ CCR6+ CD183- | 0.26 | (0.09-0.7) | 0.20 | (0.1-0.5) | 0.52 |
| Th1/Th17 cells | TCR γδ- SSC low CD4+ CCR6+ CD183+ | 0.25 | (0.1-0.6) | 0.16 | (0.07-0.3) | **0.04** |
| cTfh cells | TCR γδ- SSC low CD4+ CCR6- CXCR5- CD294+ | 2.99 | (1.9-4.1) | 2.74 | (1.8-4.9) | 0.77 |
| Ratio CD4:CD8β |  | 1.3 | (1.04-1.66) | 1.41 | (1.15-1.71) | 0.39 |
| "Regulatory T cells" panel | | | | | | |
|  | | | | | | |
| Tc cells | CD8β+ | 24.7 | (20-28) | 23.5 | (21-28) | 0.79 |
| Th cells | CD4+ | 31.7 | (27-37) | 32.6 | (27-36) | 0.93 |
| Conventional Th cells | CD4+ CD25-CD127+/CD127- | 93.4 | (90-95 | 92.6 | (91-94) | 0.22 |
| Treg cells | CD4+ CD25+ CD127- | 3.47 | (2.5-4.3) | 3.89 | (3.2-3.3) | **0.04** |
| Naive Treg cells | CD4+ CD25+ CD127- CD45RA+ HLADR - | 34.1 | (26-44) | 39.2 | (27-51) | 0.21 |
| Memory Treg cells | CD4+ CD25+ CD127- CD45RA- HLADR - | 61.3 | (50-59) | 55.4 | (42-24) | 0.14 |
| Activated Treg cells | CD4+ CD25+ CD127- CD45RA- HLADR + | 3.78 | (2.3-6.1) | 3.2 | (1.9-2.9) | 0.19 |
| Ratio CD4:CD8β |  | 1.31 | (1.05-1.65) | 1.36 | (1.09-1.66) | 0.99 |

**Table S3:** MFI (geometric mean) of circulating cells detected by flow cytometry. Results are expressed as median and interquartile range (IQR). Significant differences between stunted and non-stunted are depicted in bold letters (Mann-Whitney test).

| Variable name | Population name (Gating strategy) | Non stunted | | Stunted | | p-value |
| --- | --- | --- | --- | --- | --- | --- |
| "T cells" panel | | Median | IQR | Median | IQR |  |
| MFI CCR7 in Th CM | CD3+ CD4+ CD27+ CD45RA- CCR7+ | 35143 | (27215-41433) | 30305 | (23349-37350) | 0.07 |
| MFI CCR7 in Th EM | CD3+ CD4+ CD27- CD45RA- CCR7+ | 15484 | (12958-17211) | 13783 | (11394-16580) | 0.09 |
| MFI CCR7 in Th EMRA | CD3+ CD4+ CD27- CD45RA+ CCR7+ | 33952 | (22983-45681) | 25445 | (18358-41213) | 0.15 |
| MFI CCR7 in Th Naive | CD3+ CD4+ CD27+ CD45RA+ CCR7+ | 60588 | (47908-74057) | 53041 | (37910-67759) | 0.14 |
| MFI HLADR in Th CM | CD3+ CD4+ CD27+ CD45RA- HLADR+ | 4015 | (3527-4776) | 3515 | (3046-4299) | **0.01** |
| MFI HLADR in Th EM | CD3+ CD4+ CD27- CD45RA- HLADR+ | 3199 | (2888-3750) | 2887 | (2515-3529) | **0.05** |
| MFI HLADR in Th EMRA | CD3+ CD4+ CD27- CD45RA+ HLADR+ | 4118 | (3521-4748) | 3597 | (3216-4390) | 0.06 |
| MFI HLADR in Th Naive | CD3+ CD4+ CD27+ CD45RA+ HLADR+ | 4517 | (3928-5544) | 3995 | 3190-4881) | **0.01** |
| MFI CCR7 in Tc CM | CD3+ CD8β+ CD27+ CD45RA- CCR7+ | 14602 | (13186-16875) | 13695 | (11843-16542) | 0.15 |
| MFI CCR7 in Tc EM | CD3+ CD8β+ CD27- CD45RA- CCR7+ | 10421 | (8924-12218) | 9760 | (8220-11313) | 0.16 |
| MFI CCR7 in Tc EMRA | CD3+ CD8β+ CD27- CD45RA+ CCR7+ | 11400 | (10302-14125) | 10592 | (8877-13131) | 0.13 |
| MFI CCR7 in Tc Naive | CD3+ CD8β+ CD27+ CD45RA+ CCR7+ | 46317 | (36698-54415) | 42890 | (30261-49159) | 0.13 |
| MFI HLADR in Tc CM | CD3+ CD8β+ CD27+ CD45RA- HLADR+ | 4403 | (3726-4878) | 3796 | (3163-4550) | **0.04** |
| MFI HLADR in Tc EM | CD3+ CD8β+ CD27- CD45RA- HLADR+ | 3701 | (2972-4199) | 3182 | (2650-3963) | **0.03** |
| MFI HLADR in Tc EMRA | CD3+ CD8β+ CD27- CD45RA+ HLA-DR+ | 3074 | 2601-3635) | 2706 | 2106-3397) | **0.02** |
| MFI HLADR in Tc Naive | CD3+ CD8β+ CD27+ CD45RA+ HLA-DR+ | 4423 | (3644-5114) | 3737 | (3231-4679) | **0.01** |
| "Regulatory T cells" panel | |  |  |  |  |  |
| MFI ICOS in naive T reg | CD4+ CD25+ CD127- CD45RA+ HLA-DR- ICOS+ | 34.3 | (25-69) | 29.45 | (19-51) | 0.22 |
| MFI ICOS in memory T reg | CD4+ CD25+ CD127- CD45RA- HLA-DR- ICOS+ | 76.2 | (50-129) | 79.2 | (51-130) | 0.87 |
| MFI ICOS in activated T reg | CD4+ CD25+ CD127- CD45RA- HLA-DR+ ICOS+ | 336 | (161-594) | 322 | (191-582) | 0.74 |

**Table S4:** Multiple linear regression model summary predicting percentages of monocytes. B, unstandardized coefficient: β, standardized coefficient; SE, standard error.

|  | **Predictor** | **B** | **β** | **SE** | **t-value** | **p-value** | **p-value for model** | **Adjusted R-squared** | |
| --- | --- | --- | --- | --- | --- | --- | --- | --- | --- |
| Classical monocytes |  |  |  |  |  |  | 0.002 | 0.14 |  |
|  | Constant | 53.36 | 0.00 | 7.31 | 7.30 | 0.00 |  |  |  |
|  | Stunting (HAZ score) | 2.83 | 0.21 | 1.27 | 2.23 | 0.03 |  |  |  |
|  | Age | 0.13 | 0.09 | 0.14 | 0.92 | 0.36 |  |  |  |
|  | Sex F | -5.10 | -0.17 | 2.74 | -1.86 | 0.07 |  |  |  |
| Non-Classical monocytes |  |  |  |  |  |  | 0.02 | 0.09 |  |
|  | Constant | 13.76 | 0.00 | 4.05 | 3.40 | 0.001 |  |  |  |
|  | Stunting (HAZ score) | -1.30 | -0.18 | 0.70 | -1.85 | 0.07 |  |  |  |
|  | Age | -0.12 | -0.15 | 0.08 | -1.56 | 0.12 |  |  |  |
|  | Sex F | 2.04 | 0.13 | 1.52 | 1.34 | 0.18 |  |  |  |
| Intermediate monocytes |  |  |  |  |  |  | 0.28 | 0.02 |  |
|  | Constant | 7.36 | 0.00 | 1.78 | 4.13 | 0.0001 |  |  |  |
|  | Stunting (HAZ score) | 0.35 | 0.11 | 0.31 | 1.13 | 0.26 |  |  |  |
|  | Age | -0.02 | -0.05 | 0.03 | -0.49 | 0.63 |  |  |  |
|  | Sex F | 0.48 | 0.07 | 0.67 | 0.72 | 0.48 |  |  |  |

**Table S5:** Multiple linear regression model summary predicting expression (MFI) of HLA-DR in helper and cytotoxic T cells subsets. B, unstandardized coefficient: β, standardized coefficient; SE, standard error.

|  | **Predictor** | **B** | **β** | **SE** | **t-value** | **p-value** | **p-value for model** | **Adjusted R-squared** |
| --- | --- | --- | --- | --- | --- | --- | --- | --- |
| **MFI HLADR in Th CM** |  |  |  |  |  |  | 0.006 | 0.11 |
|  | Constant | 5760 | 0 | 716 | 8.05 | 0.000 |  |  |
|  | Stunting (HAZ score) | 119 | 0.10 | 120 | 1.00 | 0.32 |  |  |
|  | Age | -20.2 | -0.15 | 13.04 | -1.55 | 0.13 |  |  |
|  | Sex F | -461 | -0.17 | 258 | -1.79 | 0.08 |  |  |
| **MFI HLADR in Th EM** |  |  |  |  |  |  | 0.03 | 0.07 |
|  | Constant | 4253 | 0 | 487 | 8.74 | 0.000 |  |  |
|  | Stunting (HAZ score) | 106 | 0.13 | 81.6 | 1.30 | 0.20 |  |  |
|  | Age | -16.6 | -0.18 | 8.87 | -1.87 | 0.06 |  |  |
|  | Sex F | -259 | -0.14 | 176 | -1.47 | 0.14 |  |  |
| **MFI HLADR in Th EMRA** |  |  |  |  |  |  | 0.01 | 0.09 |
|  | Constant | 4846 | 0 | 661 | 7.33 | 0.000 |  |  |
|  | Stunting (HAZ score) | 155 | 0.14 | 110.8 | 1.40 | 0.16 |  |  |
|  | Age | -2.0 | -0.02 | 12.04 | -0.17 | 0.87 |  |  |
|  | Sex F | -366 | -0.14 | 239 | -1.53 | 0.13 |  |  |
| **MFI HLADR in Th Naïve** |  |  |  |  |  |  | 0.03 | 0.08 |
|  | Constant | 6403 | 0 | 917 | 6.98 | 0.000 |  |  |
|  | Stunting (HAZ score) | 218 | 0.14 | 153.8 | 1.42 | 0.16 |  |  |
|  | Age | -16.3 | -0.10 | 16.71 | -0.97 | 0.33 |  |  |
|  | Sex F | -259 | -0.07 | 331 | -0.78 | 0.44 |  |  |
| **MFI HLADR in Tc CM** |  |  |  |  |  |  | 0.003 | 0.12 |
|  | Constant | 6378 | 0 | 698 | 9.14 | 0.000 |  |  |
|  | Stunting (HAZ score) | 63 | 0.05 | 117.0 | 0.54 | 0.59 |  |  |
|  | Age | -37.7 | -0.28 | 12.71 | -2.96 | 0.00 |  |  |
|  | Sex F | -331 | -0.12 | 252 | -1.31 | 0.19 |  |  |
| **MFI HLADR in Tc EM** |  |  |  |  |  |  | 0.02 | 0.08 |
|  | Constant | 5167 | 0 | 608 | 8.50 | 0.000 |  |  |
|  | Stunting (HAZ score) | 118 | 0.11 | 101.9 | 1.16 | 0.25 |  |  |
|  | Age | -24.3 | -0.21 | 11.07 | -2.19 | 0.03 |  |  |
|  | Sex F | -345 | -0.15 | 219 | -1.57 | 0.12 |  |  |
| **MFI HLADR in Tc EMRA** |  |  |  |  |  |  | 0.02 | 0.09 |
|  | Constant | 4555 | 0 | 598 | 7.62 | 0.000 |  |  |
|  | Stunting (HAZ score) | 138 | 0.13 | 100.2 | 1.38 | 0.17 |  |  |
|  | Age | -19.2 | -0.17 | 10.89 | -1.77 | 0.08 |  |  |
|  | Sex F | -343 | -0.15 | 216 | -1.59 | 0.12 |  |  |
| **MFI HLADR in Tc Naïve** |  |  |  |  |  |  | 0.007 | 0.10 |
|  | Constant | 6461 | 0 | 885 | 7.30 | 0.000 |  |  |
|  | Stunting (HAZ score) | 148 | 0.10 | 148.3 | 1.00 | 0.32 |  |  |
|  | Age | -21.8 | -0.13 | 16.12 | -1.35 | 0.18 |  |  |
|  | Sex F | -474 | -0.14 | 319 | -1.48 | 0.14 |  |  |

**Table S6:** Multiple linear regression model summary predicting percentages of Th cells subpopulations. B, unstandardized coefficient: β, standardized coefficient; SE, standard error.

|  | **Predictor** | **B** | **β** | **SE** | **t-value** | **p-value** | **p-value for model** | **Adjusted R-squared** |
| --- | --- | --- | --- | --- | --- | --- | --- | --- |
| **Th1** |  |  |  |  |  |  | 0.10 | 0.05 |
|  | Constant | 5.39 | 0.00 | 4.89 | 1.10 | 0.27 |  |  |
|  | Stunting (HAZ score) | -0.10 | -0.01 | 0.83 | -0.12 | 0.91 |  |  |
|  | Age | 0.13 | 0.15 | 0.09 | 1.45 | 0.15 |  |  |
|  | Sex F | -0.17 | -0.01 | 1.66 | -0.10 | 0.92 |  |  |
|  | CRP | -1.47 | -0.05 | 2.78 | -0.52 | 0.60 |  |  |
|  | AAT | 0.00 | -0.01 | 0.02 | -0.11 | 0.91 |  |  |
|  | Anemia | -1.80 | -0.10 | 1.97 | -0.91 | 0.36 |  |  |
|  | *Ascaris* | -0.09 | -0.16 | 0.06 | -1.59 | 0.12 |  |  |
|  | *Encephalocytozoon* | -0.10 | -0.17 | 0.06 | -1.62 | 0.11 |  |  |
| **Th2** |  |  |  |  |  |  | 0.43 | 0.0005 |
|  | Constant | 1.26 | 0.00 | 0.70 | 1.79 | 0.08 |  |  |
|  | Stunting (HAZ score) | 0.09 | 0.08 | 0.12 | 0.75 | 0.46 |  |  |
|  | Age | 0.01 | 0.05 | 0.01 | 0.45 | 0.65 |  |  |
|  | Sex F | -0.38 | -0.16 | 0.24 | -1.59 | 0.11 |  |  |
|  | CRP | -0.58 | -0.15 | 0.40 | -1.44 | 0.15 |  |  |
|  | AAT | 0.00 | 0.17 | 0.00 | 1.50 | 0.14 |  |  |
|  | Anemia | -0.01 | 0.00 | 0.28 | -0.02 | 0.99 |  |  |
|  | *Ascaris* | 0.00 | 0.02 | 0.01 | 0.18 | 0.85 |  |  |
|  | *Encephalocytozoon* | 0.00 | 0.03 | 0.01 | 0.28 | 0.78 |  |  |
| **Th17** |  |  |  |  |  |  | 0.84 | -0.04 |
|  | Constant | 0.65 | 0.00 | 0.61 | 1.07 | 0.29 |  |  |
|  | Stunting (HAZ score) | 0.07 | 0.07 | 0.10 | 0.64 | 0.53 |  |  |
|  | Age | 0.01 | 0.07 | 0.01 | 0.60 | 0.55 |  |  |
|  | Sex F | -0.12 | -0.06 | 0.21 | -0.59 | 0.56 |  |  |
|  | CRP | -0.37 | -0.12 | 0.35 | -1.07 | 0.29 |  |  |
|  | AAT | 0.00 | -0.02 | 0.00 | -0.17 | 0.87 |  |  |
|  | Anemia | -0.04 | -0.02 | 0.24 | -0.18 | 0.86 |  |  |
|  | *Ascaris* | 0.00 | -0.02 | 0.01 | -0.21 | 0.83 |  |  |
|  | *Encephalocytozoon* | 0.00 | -0.04 | 0.01 | -0.39 | 0.70 |  |  |
| **Th1/Th17** |  |  |  |  |  |  | 0.005 | 0.13 |
|  | Constant | 0.41 | 0.00 | 0.33 | 1.25 | 0.22 |  |  |
|  | Stunting (HAZ score) | 0.05 | 0.10 | 0.06 | 0.93 | 0.35 |  |  |
|  | Age | 0.01 | 0.20 | 0.01 | 2.00 | 0.05 |  |  |
|  | Sex F | -0.16 | -0.13 | 0.11 | -1.44 | 0.15 |  |  |
|  | CRP | -0.25 | -0.13 | 0.19 | -1.31 | 0.19 |  |  |
|  | AAT | 0.00 | -0.06 | 0.00 | -0.59 | 0.55 |  |  |
|  | anemia | -0.09 | -0.07 | 0.13 | -0.71 | 0.48 |  |  |
|  | *Ascaris* | -0.01 | -0.24 | 0.00 | -2.49 | 0.01 |  |  |
|  | *Encephalocytozoon* | 0.00 | -0.02 | 0.00 | -0.20 | 0.84 |  |  |
| **cTfh** |  |  |  |  |  |  | 0.03 | 0.28 |
|  | Constant | 2.77 | 0.00 | 1.61 | 1.72 | 0.09 |  |  |
|  | Stunting (HAZ score) | -0.53 | -0.21 | 0.28 | -1.91 | 0.06 |  |  |
|  | Age | -0.01 | -0.05 | 0.03 | -0.45 | 0.65 |  |  |
|  | Sex F | 0.98 | 0.18 | 0.55 | 1.79 | 0.08 |  |  |
|  | CRP | 0.04 | 0.00 | 0.92 | 0.04 | 0.96 |  |  |
|  | AAT | 0.00 | -0.01 | 0.01 | -0.07 | 0.94 |  |  |
|  | Anemia | -0.44 | -0.07 | 0.65 | -0.69 | 0.50 |  |  |
|  | *Ascaris* | 0.00 | -0.01 | 0.02 | -0.06 | 0.95 |  |  |
|  | *Encephalocytozoon* | -0.03 | -0.17 | 0.02 | -1.63 | 0.11 |  |  |

**Table S7:** Multiple linear regression model summary predicting percentages of Treg cells subpopulations. B, unstandardized coefficient: β, standardized coefficient; SE, standard error.

|  | **Predictor** | **B** | **β** | **SE** | **t-value** | **p-value** | **p-value for model** | **Adjusted R-squared** |
| --- | --- | --- | --- | --- | --- | --- | --- | --- |
| Treg |  |  |  |  |  |  | 0.009 | 0.17 |
|  | Constant | 3.13 | 0.00 | 0.74 | 4.23 | 0.00 |  |  |
|  | Stunting (HAZ score) | -0.29 | -0.24 | 0.12 | -2.37 | 0.02 |  |  |
|  | Age | 0.00 | 0.01 | 0.01 | 0.14 | 0.89 |  |  |
|  | Sex F | -0.38 | -0.14 | 0.26 | -1.48 | 0.14 |  |  |
| Treg naïve |  |  |  |  |  |  | 0.000 | 0.29 |
|  | Constant | 31.31 | 0.00 | 7.53 | 4.16 | 0.00 |  |  |
|  | Stunting (HAZ score) | -3.22 | -0.23 | 1.26 | -2.56 | 0.01 |  |  |
|  | Age | -0.25 | -0.17 | 0.13 | -1.93 | 0.06 |  |  |
|  | Sex F | 5.25 | 0.17 | 2.60 | 2.01 | 0.05 |  |  |
| Treg memory | |  |  |  |  |  | 0.003 | 0.14 |
|  | Constant | 64.69 | 0.00 | 8.14 | 7.95 | 0.00 |  |  |
|  | Stunting (HAZ score) | 2.86 | 0.21 | 1.36 | 2.10 | 0.04 |  |  |
|  | Age | 0.10 | 0.07 | 0.14 | 0.70 | 0.49 |  |  |
|  | Sex F | -6.06 | -0.20 | 2.82 | -2.15 | 0.03 |  |  |
| Treg activated | |  |  |  |  |  | 0.511 | -0.01 |
|  | Constant | 4.66 | 0.00 | 1.50 | 3.11 | 0.00 |  |  |
|  | Stunting (HAZ score) | 0.44 | 0.19 | 0.25 | 1.75 | 0.08 |  |  |
|  | Age | 0.01 | 0.05 | 0.03 | 0.48 | 0.64 |  |  |
|  | Sex F | -0.13 | -0.03 | 0.52 | -0.26 | 0.80 |  |  |

**Table S8**: CRP data from the 105 selected children.

| ID | CRP_value | CRP_threshold | ID | CRP_value | CRP_threshold |
| --- | --- | --- | --- | --- | --- |
| 1 | <06.0 | Normal (<=10 mg/l) | **54** | 10 | Normal (<=10 mg/l) |
| 2 | <06.0 | Normal (<=10 mg/l) | **55** | 6 | Normal (<=10 mg/l) |
| 3 | <06.0 | Normal (<=10 mg/l) | **56** | <06.0 | Normal (<=10 mg/l) |
| 4 | <06.0 | Normal (<=10 mg/l) | **57** | <06.0 | Normal (<=10 mg/l) |
| 5 | <06.0 | Normal (<=10 mg/l) | **58** | 22 | High (>10 mg/l) |
| 6 | <06.0 | Normal (<=10 mg/l) | **59** | <06.0 | Normal (<=10 mg/l) |
| 7 | <06.0 | Normal (<=10 mg/l) | **60** | <06.0 | Normal (<=10 mg/l) |
| 8 | 6 | Normal (<=10 mg/l) | **61** | <06.0 | Normal (<=10 mg/l) |
| 9 | <06.0 | Normal (<=10 mg/l) | **62** | 7 | Normal (<=10 mg/l) |
| 10 | <06.0 | Normal (<=10 mg/l) | **63** | <06.0 | Normal (<=10 mg/l) |
| 11 | <06.0 | Normal (<=10 mg/l) | **64** | <06.0 | Normal (<=10 mg/l) |
| 12 | <06.0 | Normal (<=10 mg/l) | **65** | <06.0 | Normal (<=10 mg/l) |
| 13 | <06.0 | Normal (<=10 mg/l) | **66** | <06.0 | Normal (<=10 mg/l) |
| 14 | <06.0 | Normal (<=10 mg/l) | **67** | <06.0 | Normal (<=10 mg/l) |
| 15 | <06.0 | Normal (<=10 mg/l) | **68** | <06.0 | Normal (<=10 mg/l) |
| 16 | <06.0 | Normal (<=10 mg/l) | **69** | <06.0 | Normal (<=10 mg/l) |
| 17 | <06.0 | Normal (<=10 mg/l) | **70** | <06.0 | Normal (<=10 mg/l) |
| 18 | <06.0 | Normal (<=10 mg/l) | **71** | <06.0 | Normal (<=10 mg/l) |
| 19 | <06.0 | Normal (<=10 mg/l) | **72** | <06.0 | Normal (<=10 mg/l) |
| 20 | <06.0 | Normal (<=10 mg/l) | **73** | <06.0 | Normal (<=10 mg/l) |
| 21 | <06.0 | Normal (<=10 mg/l) | **74** | <06.0 | Normal (<=10 mg/l) |
| 22 | 7 | Normal (<=10 mg/l) | **75** | <06.0 | Normal (<=10 mg/l) |
| 23 | 9 | Normal (<=10 mg/l) | **76** | 14 | High (>10 mg/l) |
| 24 | <06.0 | Normal (<=10 mg/l) | **77** | <06.0 | Normal (<=10 mg/l) |
| 25 | 11 | High (>10 mg/l) | **78** | <06.0 | Normal (<=10 mg/l) |
| 26 | <06.0 | Normal (<=10 mg/l) | **79** | <06.0 | Normal (<=10 mg/l) |
| 27 | <06.0 | Normal (<=10 mg/l) | **80** | 13 | High (>10 mg/l) |
| 28 | <06.0 | Normal (<=10 mg/l) | **81** | 14 | High (>10 mg/l) |
| 29 | <06.0 | Normal (<=10 mg/l) | **82** | 19 | High (>10 mg/l) |
| 30 | <06.0 | Normal (<=10 mg/l) | **83** | <06.0 | Normal (<=10 mg/l) |
| 31 | <06.0 | Normal (<=10 mg/l) | **84** | <06.0 | Normal (<=10 mg/l) |
| 32 | <06.0 | Normal (<=10 mg/l) | **85** | <06.0 | Normal (<=10 mg/l) |
| 33 | <06.0 | Normal (<=10 mg/l) | **86** | 11 | High (>10 mg/l) |
| 34 | <06.0 | Normal (<=10 mg/l) | **87** | <06.0 | Normal (<=10 mg/l) |
| 35 | <06.0 | Normal (<=10 mg/l) | **88** | <06.0 | Normal (<=10 mg/l) |
| 36 | <06.0 | Normal (<=10 mg/l) | **89** | <06.0 | Normal (<=10 mg/l) |
| 37 | <06.0 | Normal (<=10 mg/l) | **90** | <06.0 | Normal (<=10 mg/l) |
| 38 | 10 | Normal (<=10 mg/l) | **91** | <06.0 | Normal (<=10 mg/l) |
| 39 | <06.0 | Normal (<=10 mg/l) | **92** | <06.0 | Normal (<=10 mg/l) |
| 40 | <06.0 | Normal (<=10 mg/l) | **93** | <06.0 | Normal (<=10 mg/l) |
| 41 | 5 | Normal (<=10 mg/l) | **94** | <06.0 | Normal (<=10 mg/l) |
| 42 | <06.0 | Normal (<=10 mg/l) | **95** | <06.0 | Normal (<=10 mg/l) |
| 43 | <06.0 | Normal (<=10 mg/l) | **96** | <06.0 | Normal (<=10 mg/l) |
| 44 | <06.0 | Normal (<=10 mg/l) | **97** | 9 | Normal (<=10 mg/l) |
| 45 | <06.0 | Normal (<=10 mg/l) | **98** | 20 | High (>10 mg/l) |
| 46 | <06.0 | Normal (<=10 mg/l) | **99** | <06.0 | Normal (<=10 mg/l) |
| 47 | <06.0 | Normal (<=10 mg/l) | **100** | 17 | High (>10 mg/l) |
| 48 | <06.0 | Normal (<=10 mg/l) | **101** | <06.0 | Normal (<=10 mg/l) |
| 49 | <06.0 | Normal (<=10 mg/l) | **102** | 11 | High (>10 mg/l) |
| 50 | <06.0 | Normal (<=10 mg/l) | **103** | 8 | Normal (<=10 mg/l) |
| 51 | 24 | High (>10 mg/l) | **104** | <06.0 | Normal (<=10 mg/l) |
| 52 | <06.0 | Normal (<=10 mg/l) | **105** | <06.0 | Normal (<=10 mg/l) |
| 53 | 12 | High (>10 mg/l) |  |  |  |

## Supplementary Figures

**
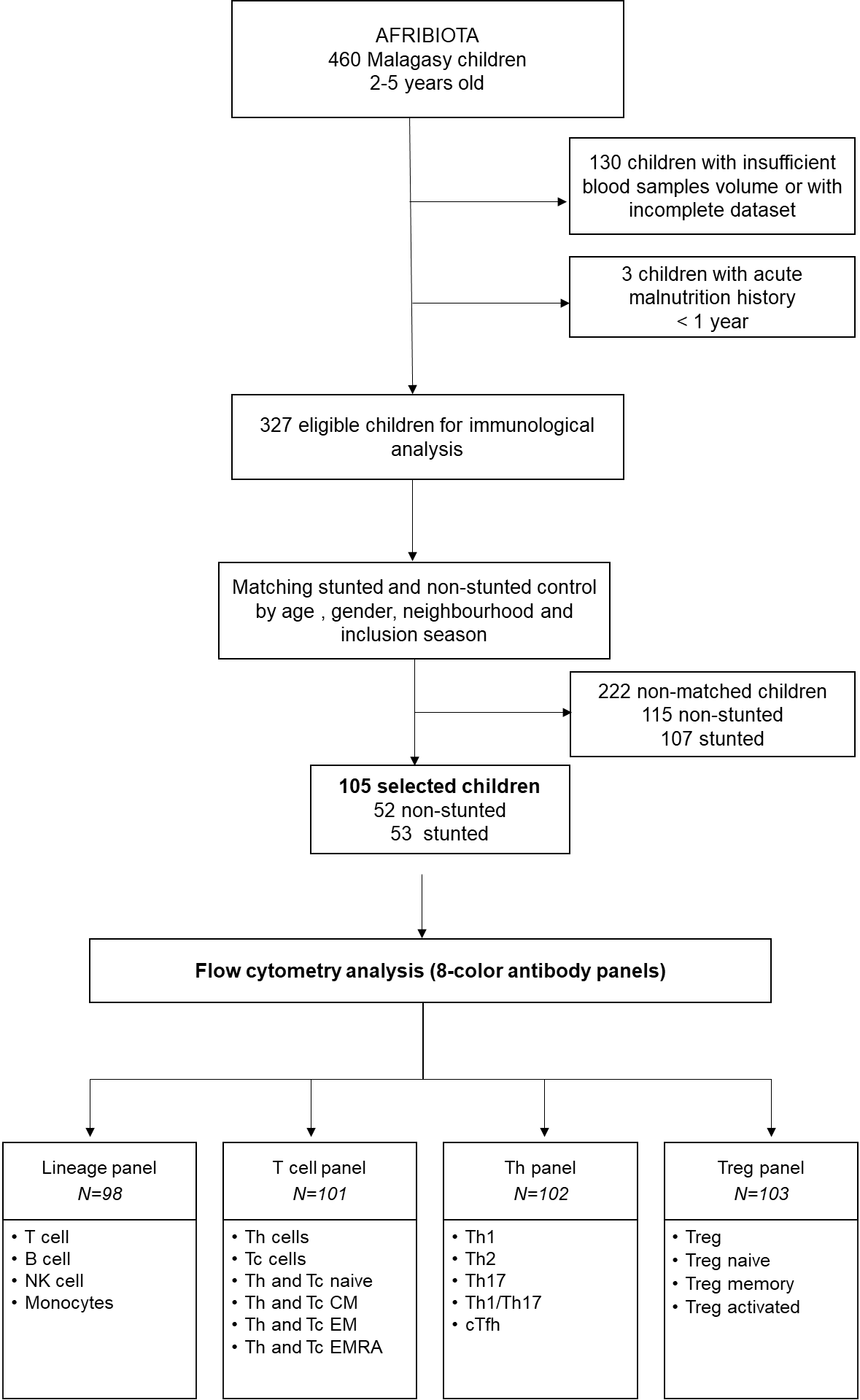
**

**Fig S1: Flowchart of children included in the study.** HIV-negative children aged 2 to 5 years, neither suffering from acute malnutrition, nor from any other severe disease, recruited in Antananarivo (Madagascar), were selected for this study. Children with enough blood samples and complete epidemiological data set were selected for immunological analysis. Immunophenotyping and flow cytometry analysis were performed on cryopreserved PBMC using 8-coulor antibody panels. Several subsets of T lymphocytes were analysed: CM; Central Memory, EM; Effector Memory, EMRA; Effector Memory CD45RA+, Th1: T helper 1, Th2: T helper 2, Th17: T helper 17, cTfh: circulating T follicular helper and Treg; Regulatory T cells.

**
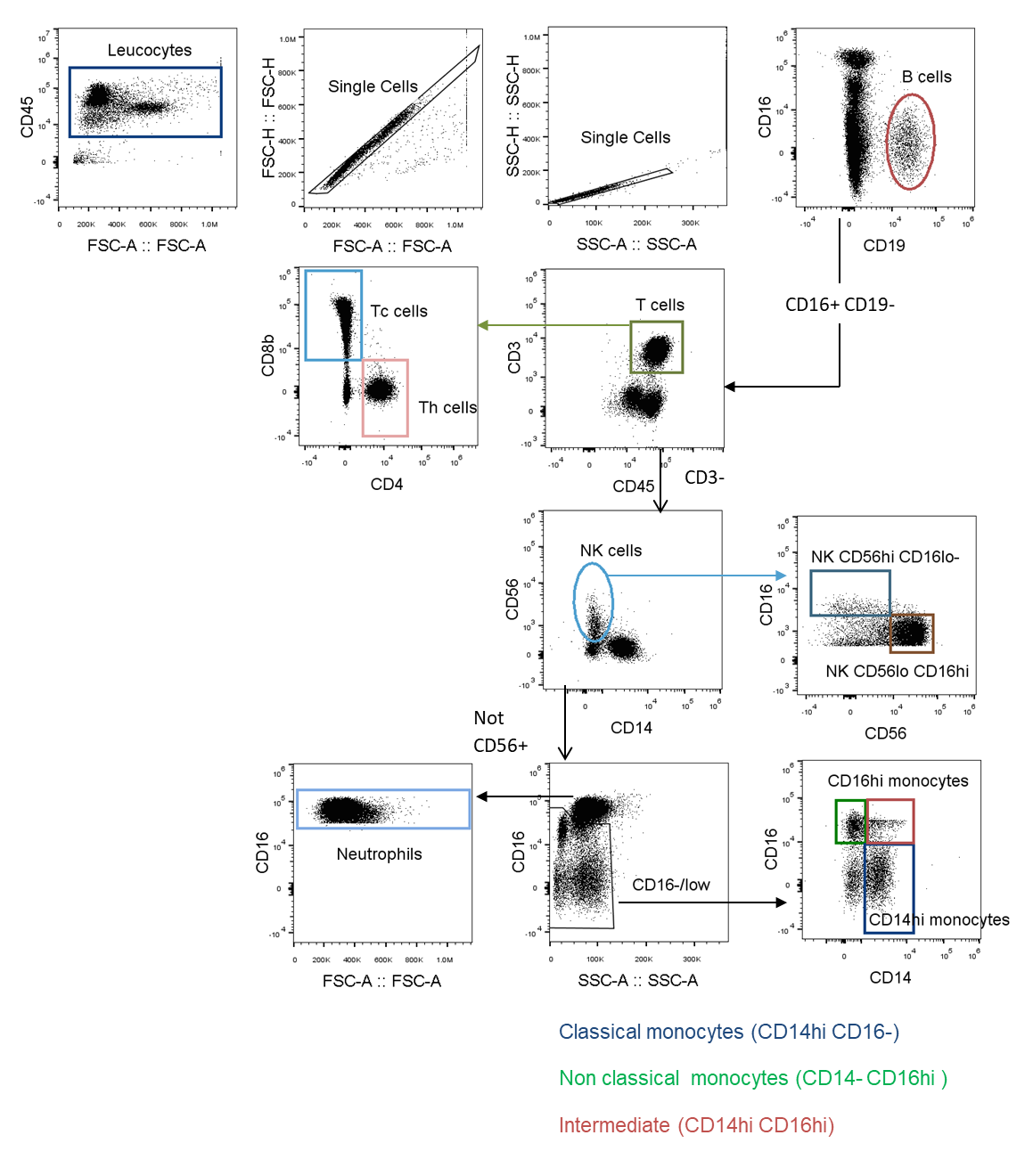
**

**Fig S2: Gating strategy for the “Lineage panel”.** Leucocytes were identified by CD45 expression. Live cells were analysed through sequential singlet gating. B cells were identified as CD19^+^CD16^-^ and T cells were gated as CD19^-^ cells followed by CD3^+^ staining. CD4^+^ cells and CD8^+^ cells within the T cells population were gated. Within the CD3^-^ cells, NK cells were identified as CD56^+^ and analysed for their expression of CD16 and CD56. In the population of CD56^-^ cells, CD16^hi^SSC^low^ cells were selected to separate monocytes from neutrophils. Further gating identified classical (CD14^hi^CD16^-^), non-classical (CD14^-^CD16^hi^) and intermediate (CD14^hi^CD16^hi^) monocytes. Neutrophils were defined as CD16^hi^FSC^hi^.


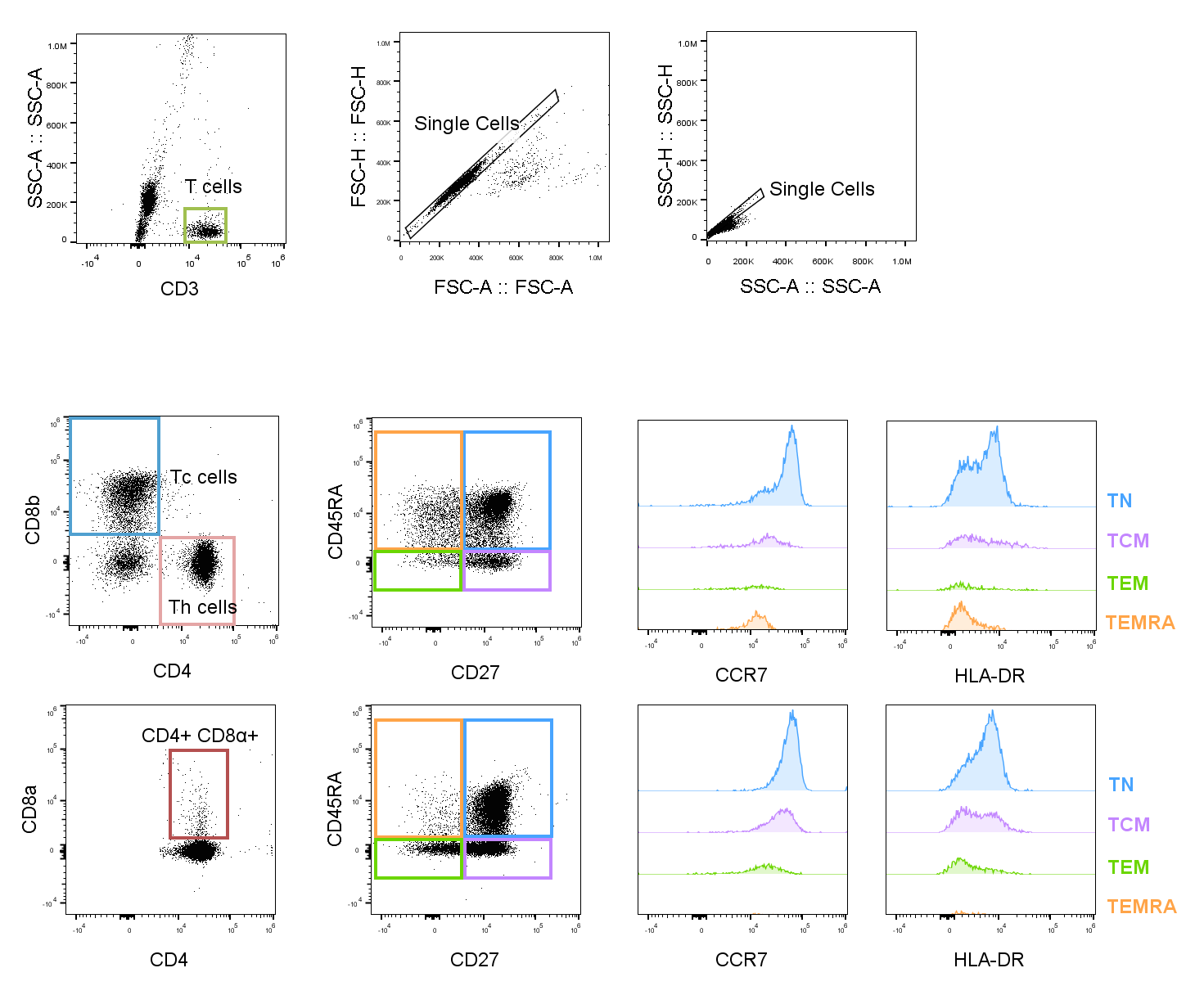


**Fig. S3: Gating strategy for the general “T cell panel”.** T cells were identified as CD3^+^ cells. Upon exclusion of doublets, CD4^+^ and CD8β^+^ were gated. Based on their expression of CD45RA and CD27, T cell subpopulations were identified as naïve (TN), central memory (TCM), effector memory (TEM) and effector memory expressing RA (TEMRA). Expressions of CCR7 and HLA-DR were determined and plotted as a histogram. The CD4^+^CD8β^−^ cells expressing CD8α were also identified.


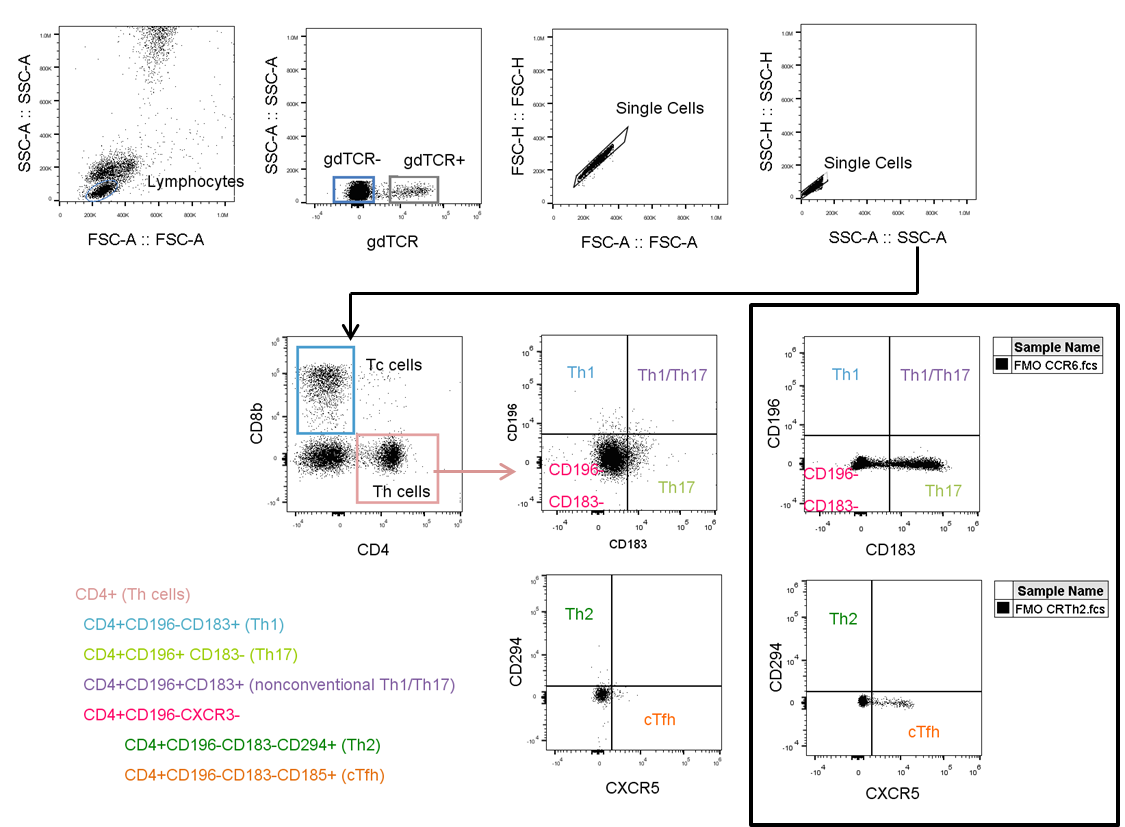


**Fig. S4: Gating strategy for “T helper cell panel”.** Lymphocytes populations were gated with FSC and SSC scatter. Doublets were excluded using FSC-W/FSC-H and SSC-A/SSC-H parameters. Th cells were identified as γδTCR^-^CD4^+^ and Tc cells as γδTCR^-^CD8^+^. Using CD196, CD183, CXCR5 and CD294 expressions, Th cells subsets were identified as Th1 (CD183^+^CCR6^-^), Th17 (CD183^-^CCR6^+^), Th1Th17 (CD183^+^CCR6^+^CD183^+^CD194^+^), Th2 (CD183^-^CCR6^-^CD294^+^CXCR5^-^) and cTfh (CD294^-^CXCR5^+^). FMO controls were prepared for CCR6 and CRTh2 (CD294).


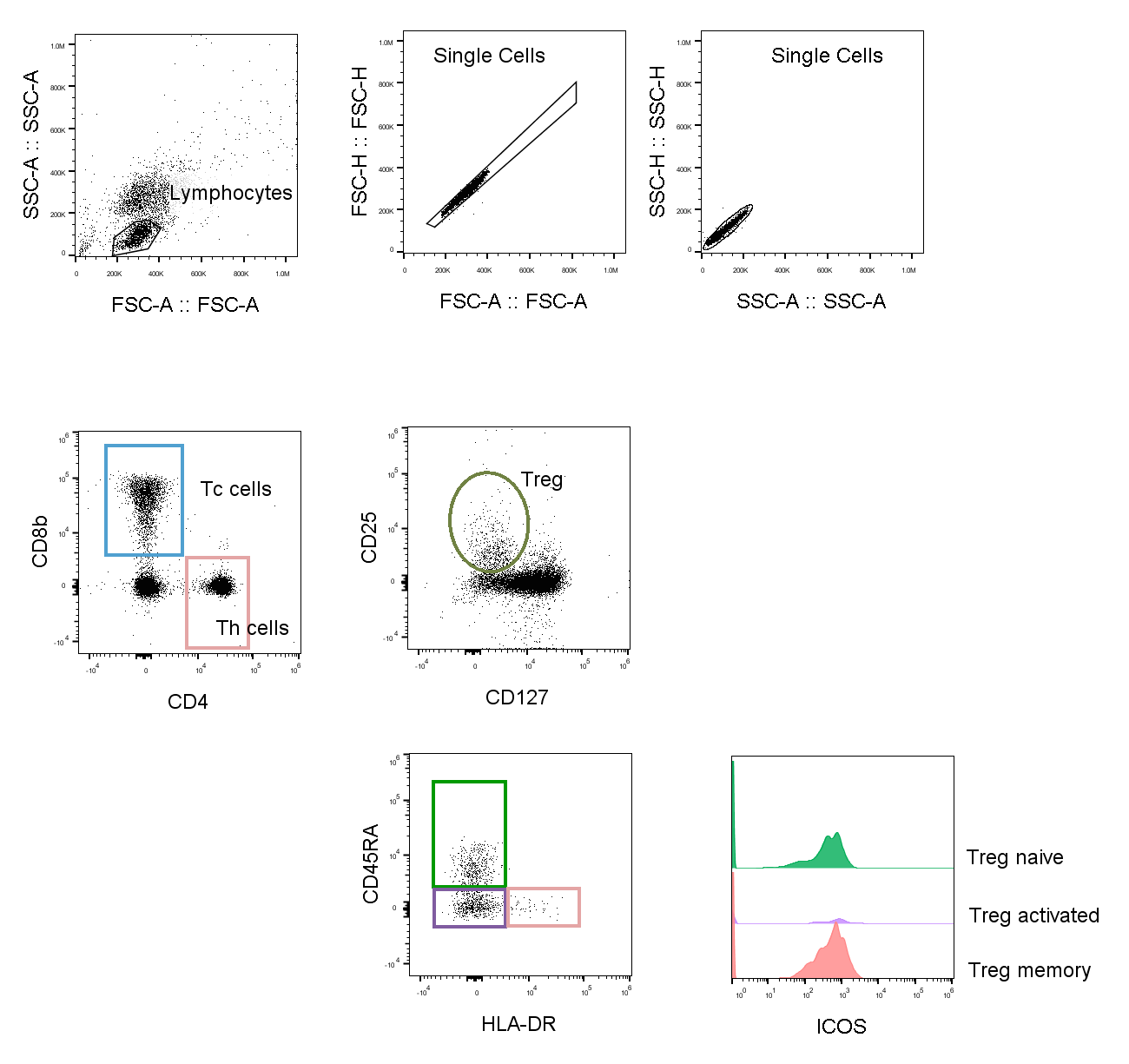


**Fig. S5: Gating strategy for the “T regulatory cell panel”**. Lymphocytes were gated by FSC vs. SSC, followed by sequential elimination of doublets and CD4^+^/CD8β T cell gating. CD25^+^CD127^-^ Tregs were gated out of CD4^+^ T cells. Treg cells subpopulations were identified with CD45RA and HLA-DR expressions as naïve Treg (CD45RA^+^HLA-DR^-^), memory Treg (CD45RA^-^HLA-DR^-^) and activated Treg (CD45RA^-^HLA-DR^+^). Relative fluorescence of ICOS was calculated and plotted as histogram in the Treg subsets.


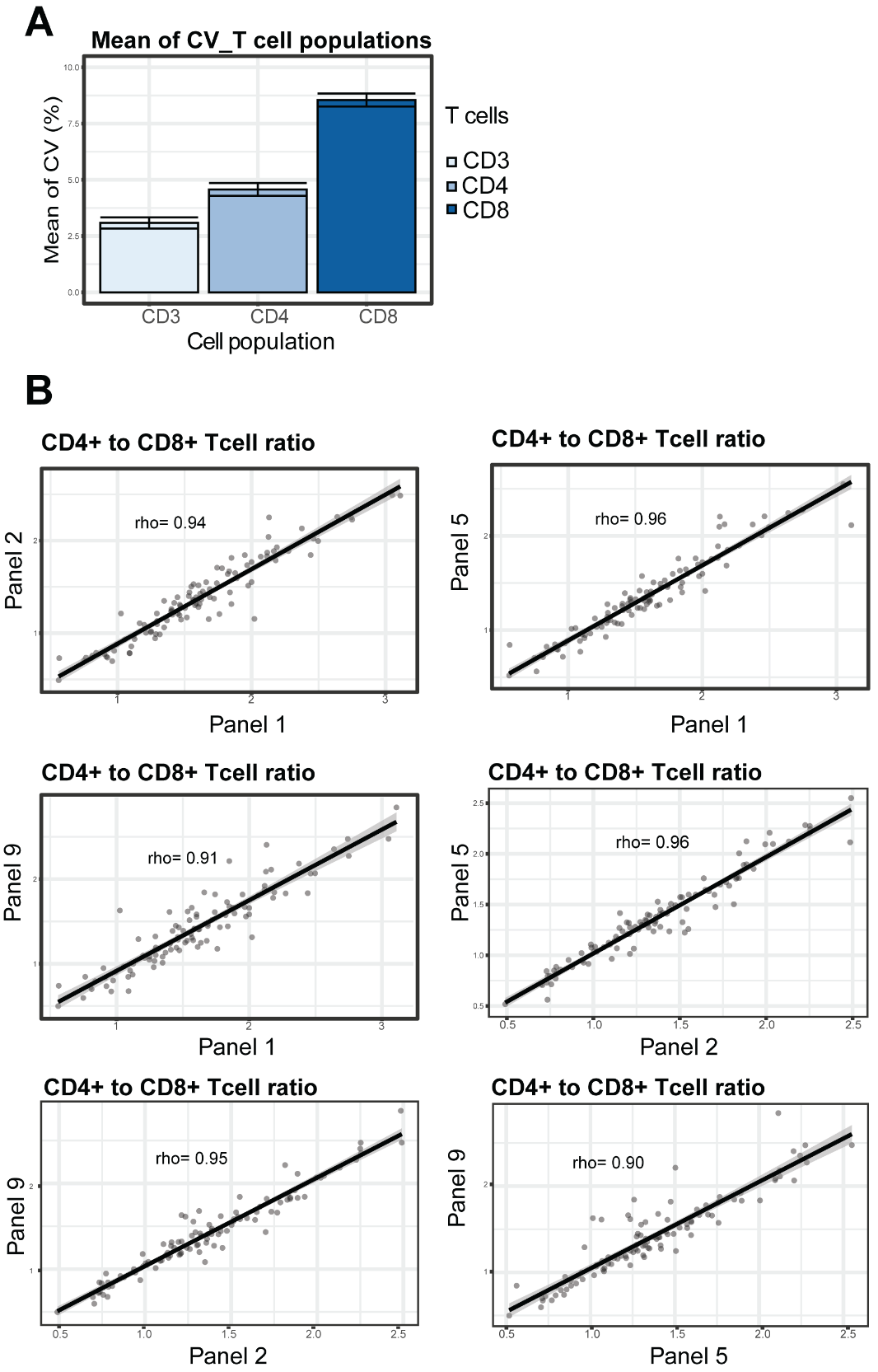


**Fig S6:** **Variability of T cells measurements across panels and individuals.** **(A)** Mean of coefficient of variations of T cells subsets frequencies: CD3+ between “lineage panel” and “T cell panel”; CD4+, and CD8+ populations between the 4 panels. **(B)** Comparison of CD4+ to CD8+ ratio measured in the 4 panels. Panel 1: “T cell panel”, Panel 2: “Treg panel”, Panel 5: “Lineage panel”, Panel 9: “Th panel”. Spearman’s correlation coefficients (rho) are shown.


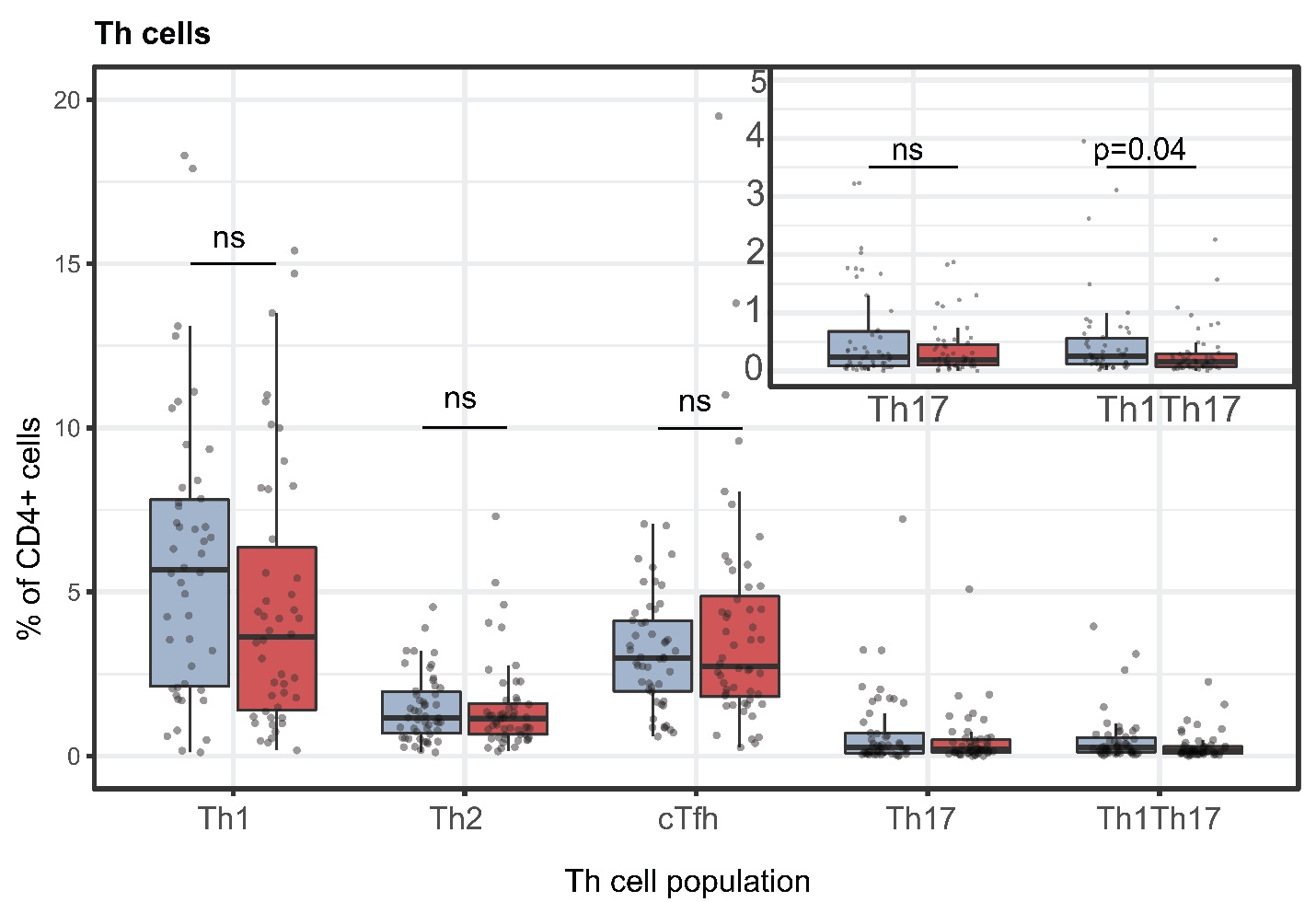


**Fig S7:** **Frequencies of T helper cell subsets percentages in stunted versus non-stunted children**. P-values were calculated using Wilcoxon rank-sum test and significance was fixed at p<0.05, ns: not significant.


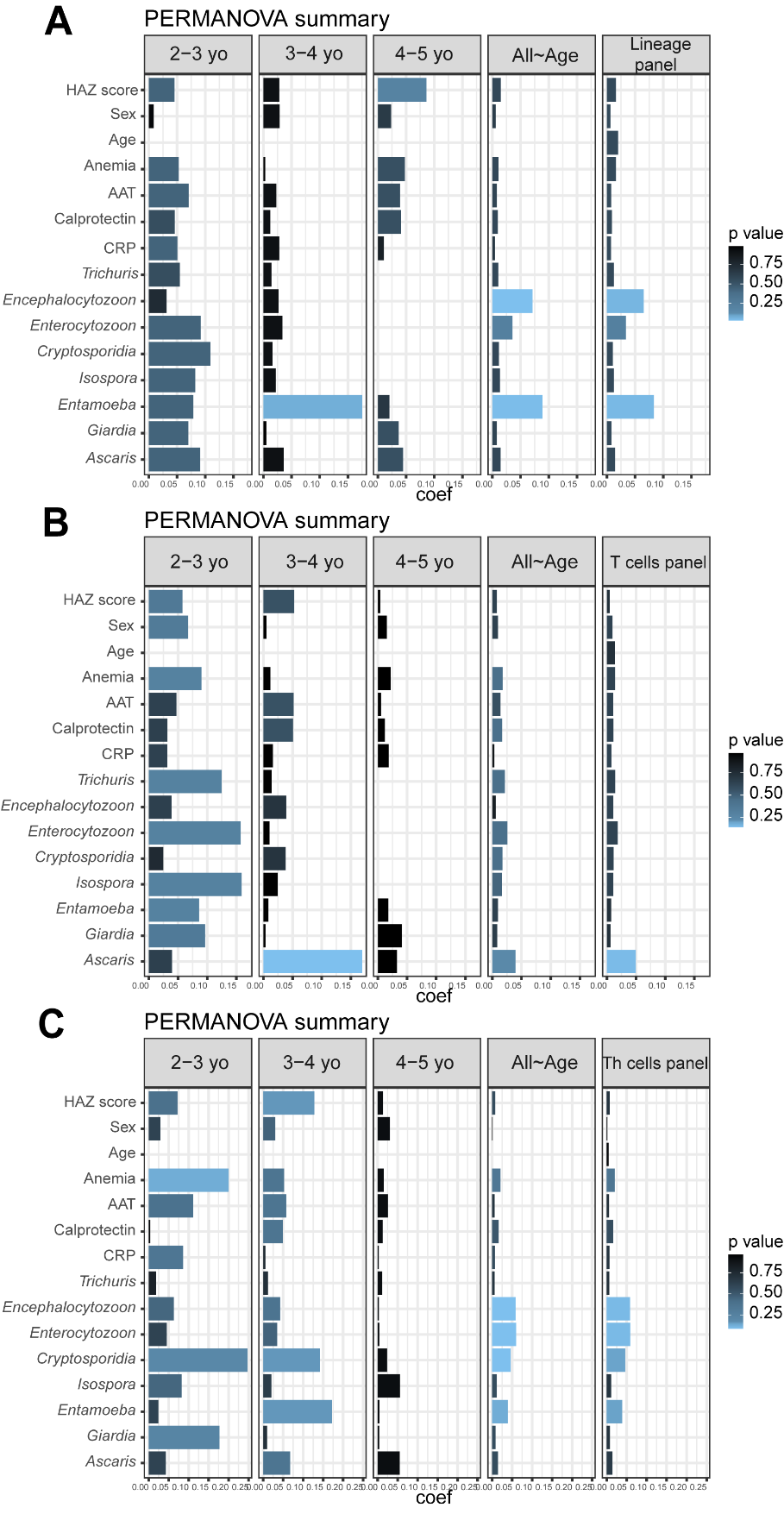


**Fig. S8:** **Distribution of cell percentages in the “lineage panel” (A), “T cells panel” (B) and “T helper cells panel” (C).** Summary of PERMANOVA analysis of the cell percentages in the full dataset and stratified by age and in each age category individually. Analysis is based on the non-overlapped populations in each panel. “Panel 5: Lineage panel”: Tc cells, Th cells, NK cells CD56^high^, NK cells CD56^low^, monocytes CD14 ^high^, monocytes CD16^high^, neutrophils. “Panel 1: T cells panel”: T cells CD8α+, Th cells Central memory, Th cells EM, Th cells EMRA, Th cells Naïve, Tc cells CM, Tc cells EM, Tc cells EMRA, Tc cells Naive. “Panel 9: T helper cells panel”: Tc cells CCR6+ CD183-, Tc cells CCR6+ CD183+ Tc cells CCR6-CD183+, Tc cells CCR6-CD183-, Th1 cells, Th2 cells, Th17 cells, Th1/Th17 cells, cTfh cells. Each variable was tested individually in the PERMANOVA without other covariates. Coef: the coefficient of variance by PERMANOVA. The tested variables were as follows: Stunting status (height-for-age z-score); Age (child’s age in months); Anemia (presence or absence of anemia); AAT (fecal alpha-1 antitrypsin in mg/g dry weight); Calprotectin (fecal calprotectin in μg/g dry weight); CRP (serum C-reactive protein in mg/l); and parasite carriage. *N* total= 105, *N* 2 years old= 26, *N* 3 years old= 36, *N* 4-5 years old= 40.
